# Trends and Variations in Emergency Department Use Associated With Diabetes in the US by Sociodemographic Factors, 2008-2017

**DOI:** 10.1001/jamanetworkopen.2022.13867

**Published:** 2022-05-25

**Authors:** Tegveer S. Uppal, Puneet Kaur Chehal, Gail Fernandes, J. Sonya Haw, Megha Shah, Sara Turbow, Swapnil Rajpathak, K. M. Venkat Narayan, Mohammed K. Ali

**Affiliations:** 1 Hubert Department of Global Health, Rollins School of Public Health, Emory University, Atlanta, Georgia; 2 Department of Health Policy and Management, Rollins School of Public Health, Emory University, Atlanta, Georgia; 3 Merck & Co Inc, Kenilworth, New Jersey; 4 Division of Endocrinology and Metabolism, Department of Medicine, School of Medicine, Emory University, Atlanta, Georgia; 5 Department of Family and Preventive Medicine, School of Medicine, Emory University, Atlanta, Georgia; 6 Division of General Medicine and Geriatrics, Department of Medicine, School of Medicine, Emory University, Atlanta, Georgia

## Abstract

**Importance:**

Little is known about emergency department (ED) use among people with diabetes and whether the pattern of ED use varies across geographic areas and population subgroups.

**Objective:**

To estimate recent national- and state-level trends in diabetes-related ED use overall and by race and ethnicity, rural or urban location, and insurance status.

**Design, Setting, and Participants:**

This cross-sectional study of adults visiting the ED with a diabetes-related diagnosis used serial data from the Nationwide Emergency Department Sample, a nationally representative database, and discharge records from 11 state emergency department databases for 2008, 2011, 2014, and 2016 to 2017. Data were analyzed from March 16 to November 9, 2020.

**Exposures:**

Reported race and ethnicity, rural or urban location, and insurance status. Data were stratified to generate state-specific estimates.

**Main Outcomes and Measures:**

Rates of ED use for all-cause visits among adults with diabetes (all-cause diabetes visits) and visits with primary diagnoses of diabetes-specific complications.

**Results:**

A larger portion of all-cause diabetes ED visits (n = 32 433 015) were by female (56.8%) and middle-aged (mean [SD] age, 58.4 [16.3] years) adults with diabetes. Nationally, all-cause diabetes ED visits per 10 000 adults increased 55.6% (95% CI, 50.6%-60.6%), from 257.6 (95% CI, 249.9-265.3) visits in 2008 to 400.8 (95% CI, 387.6-414.0) visits in 2017. All-cause diabetes ED visits increased more for urban (58.3%; 95% CI, 52.5%-64.1%) and uninsured subgroups (75.3% [95% CI, 59.8%-90.8%]) than for their counterparts. Diabetes-specific ED visits (weighted number of 1 911 795) nationally increased slightly among all subgroups. State-specific ED use rates show wide state-to-state variations in ED use by race and ethnicity, rural or urban location, and insurance. On average across states, diabetes-specific ED use among Black patients was approximately 3 times (rate ratio, 3.09 [95% CI, 2.91-3.30]) greater than among non-Hispanic White patients, and among Hispanic patients, it was 29% greater (rate ratio, 1.29 [95% CI, 1.19-1.40]) than among non-Hispanic White patients. The mean rate of ED use among rural patients was 34% greater (rate ratio, 1.34 [95% CI, 1.26-1.44]) than among urban patients. The mean rates of ED use among patients with Medicaid (rate ratio, 6.65 [95% CI, 6.49-6.82]) and Medicare (rate ratio, 4.37 [95% CI, 4.23-4.51]) were greater than among privately insured adults.

**Conclusions and Relevance:**

This study suggests that disparities in diabetes-related ED use associated with race and ethnicity, rural or urban location, and insurance status were persistent from 2008 to 2017 within and across states, as well as nationally. Further geographic and demographic-specific analyses are needed to understand the sources of inequity.

## Key Points

**Question:**

Has emergency department (ED) use for diabetes-related illness changed over time in the United States, and does it vary by state, level of urbanization, race and ethnicity, and insurance type?

**Findings:**

In this serial cross-sectional study of 32 million ED visits from 2008 to 2017, the rates of diabetes-related ED use increased across all geographic areas and subgroups studied. Racial and ethnic disparities and rural and urban disparities in ED use varied widely across states and remained significantly different within states, with Black adults having a mean of approximately 3 times as many diabetes-specific ED visits as White adults.

**Meaning:**

This study suggests that, despite health reforms during the past decade, disparities in diabetes-related ED use persisted from 2008 to 2017, warranting further study and policy action to address access and underlying social determinants of health.

## Teaser

This cross-sectional study uses data from the Nationwide Emergency Department Sample to estimate recent national- and state-level trends in diabetes-related emergency department use overall and by race and ethnicity, rural or urban location, and insurance status.

## Introduction

From 1990 to 2018, the number of US patients with diagnosed diabetes more than quadrupled, from 6.5 million to 26.8 million.^1,2^ Diabetes bears large costs to society; the annual direct costs amount to $237 billion, representing approximately 1 in 4 health care dollars spent in the United States,^3^ with greater costs observed in racial and ethnic minority groups.^4^

Reports have also highlighted wide-ranging disparities in the prevalence of diabetes,^5,6^ the quality of diabetes-related care,^7,8^ and outcomes.^9,10,11^ Black and Hispanic patients account for a disproportionate share of diabetes complications and worse disease-related outcomes.^12,13^ Furthermore, rural areas report a disproportionate burden of diabetes-related morbidity and mortality.^14,15^

In addition to the increased prevalence of diabetes, there have been several significant social and political events that likely were associated with the incidence and management of diabetes through social determinants of health and access to care; for example, the Great Recession, the Patient Protection and Affordable Care Act (ACA), and accelerating rural hospital closures.^16,17,18,19^ Because emergency department (ED) use is recognized as a proxy for lack of access to care, quantifying trends in diabetes-related ED use provides an assessment of the fragmented US health care system’s ability to address the increased prevalence of diabetes.^20,21,22^

To our knowledge, the existing literature on the trends in ED use, using high-quality administrative data, does not distinguish between diabetes-related ED use and other reasons for ED use,^23^ focuses on specific select diabetes complications,^24,25,26^ and reports dated estimates of diabetes-related ED use.^27^ The recent trends in aggregate diabetes-related ED use and the variations within and across geographic and sociodemographic subgroups remain unclear. We used all-payer data to explore whether disparities in diabetes-related ED use associated with race and ethnicity, rural and urban location, and insurance status have changed over time, and we examined national, within-state, and between-state heterogeneity.

## Methods

### Data Sources

To estimate national diabetes-related ED visits, we used data from the Healthcare Cost and Utilization Project (HCUP) Nationwide Emergency Department Sample (NEDS) of the Agency for Healthcare Research and Quality’s (AHRQ). We also used a subset of data from the State Emergency Department Databases (SEDD) to estimate state-specific diabetes-related ED visits.^28,29^ We used NEDS and SEDD data for the years 2008, 2011, 2014, and 2016 to 2017 (2017 data for Vermont, New York, and the NEDS were not available during data acquisition).

The NEDS is a 20% stratified sample of ED discharges from hospitals participating in HCUP and is the largest all-payer ED database in the United States. NEDS data are weighted to provide nationally representative estimates of ED visits. The data from SEDD are derived from a census of hospitals for a state for a given year and contain discharge records of all ED visits.^29^ We analyzed data from Arizona, Florida, Iowa, Kentucky, Maryland, Nebraska, New Jersey, New York, North Carolina, Utah, and Vermont. All states participated as HCUP partners for the whole study period and were selected to maximize the mix of demographically and geographically diverse states. This study was deemed exempt by the Emory University Institutional Review Board because it was not human participants research. This study followed the Strengthening the Reporting of Observational Studies in Epidemiology (STROBE) reporting guideline.^30^

We used data from the American Community Survey to estimate population denominators for corresponding years of the HCUP data by demographic and insurance subgroups for state and national populations to calculate ED visit rates.^31,32^ The American Community Survey is conducted annually by the US Census Bureau and includes information about respondent demographic, social, economic, and housing characteristics; a mean of 2.15 million households are surveyed each year.^31,33^

### Study Population

We selected NEDS and SEDD discharge records (eFigure 1 and eFigure 2 in the Supplement) for (1) adult patients (≥18 years), (2) discharges not resulting in admission, and (3) presence of *International Classification of Diseases, Ninth Revision* (*ICD-9*) or *International Statistical Classification of Diseases and Related Health Problems, Tenth Revision* (*ICD-10*) codes indicative of type 1 or 2 diabetes (*ICD-9* code 250.XX or *ICD-10* codes E10.XXX, E11.XXX, and E13.XXX).^34^ Records for patients with gestational or secondary diabetes were excluded. We excluded patients with severe illness (discharges that resulted in admission) to select for “treat-and-release” visits, which are highly likely to be preventable through diligent outpatient management.^23^

### Outcomes

We referred to ED visits for any clinical reason by individuals with diabetes as “all-cause diabetes ED visits,” which included ED discharges for non–diabetes-related reasons when there was a diabetes diagnosis on the discharge record. Emergency department visits by individuals with diabetes for diabetes-specific complications were referred to as “diabetes-specific ED visits,” defined by presence of a principal diagnosis for a diabetes-specific condition (short-term diabetes complications [eg, ketoacidosis and hyperosmolarity], long-term diabetes complications [eg, kidney and eye complications], uncontrolled diabetes, and limb ulcers or inflammation). Definitions were derived from the AHRQ’s list of ambulatory care–sensitive conditions, which are complications that are preventable with access to high-quality outpatient care.^35,36^ Complete *ICD-9* and *ICD-10* code lists are in eTable 4 in the Supplement.

### Subgroups

Subgroup-specific numerators were estimated for hospital-reported race and ethnicity (Black, White, and Hispanic), rural vs urban patient location, and insurance (Medicaid, Medicare, privately insured, and uninsured). Race and ethnicity were recorded by hospital staff on patient discharge records. There is likely hospital-level variation in the race and ethnicity data collection processes. State-level HCUP data partners compiled these administrative hospital data and provided linkage and validation services prior to providing data to HCUP. The funding agency did require race and ethnicity variables. We assessed race and ethnicity owing to existing evidence of disparities for patients with diabetes.

National estimates were also stratified by region (Midwest, Northeast, South, and West) but not by race and ethnicity because the NEDS does not provide a race variable. We selected complete records for all analytic variables with less than 1% missing data for both national and state data sets. Individual SEDD yearly data sets missing 1% or more of data on any of the analytic variables were imputed using the MICE package in R, version 4.0.0 (R Group for Statistical Computing).^37^ A full description of missing data can be found in eTables 1, 2, and 3 in the Supplement, and the methods for addressing missing data can be found in eAppendix 1 in the Supplement.

### Statistical Analysis

#### Estimating Numerators and Measures of Uncertainty

Statistical analysis was performed using R, version 4.0.0 from March 16 to November 9, 2020. The numbers of ED visits generated using NEDS were weighted and adjusted for complex survey design using weighting variables provided by HCUP.^38^ We calculated SEs and variance for survey-weighted estimates using Taylor Series Linearization methods provided in the *Survey* package for R, version 4.0.0.^39^

The numbers of diabetes-related ED visits generated using SEDD data were aggregated because the data represent a census of all visits to the ED in each state.^29^ We assumed a Poisson distribution for the purpose of estimating the variance in the count estimates.^40,41^

#### Rate Calculation, Standardization, and Reporting

We estimated overall ED visit rates (both nationally and by state) per 10 000 persons per year, using corresponding survey-weighted denominator estimates from the American Community Survey. Population estimates for racial and ethnic, rural and urban, and regional subgroups were age-standardized to the 2010 US adult population using the direct method to facilitate subgroup comparisons across states and years by accounting for varying age distributions. More information on denominator population estimates and calculations is available in eAppendix 2 in the Supplement. As a sensitivity analysis, we recalculated rates using denominator data available from the Behavioral Risk Factor Surveillance System Study to examine trends in use by diabetes status (eAppendix 3 in the Supplement).^42^ We calculated rate ratios to compare rates of ED use between sociodemographic groups and by diabetes status.

We report demographic-specific and insurance-specific rates by year nationally and by state, between-group rate ratios, and percentage changes in rates over 2008 to 2016 and 2017. Plots were generated in R using the *ggplot2* package.^43^

## Results

Nationally, for 2008, 2011, 2014, and 2016 and 2017, we identified 32 433 015 all-cause ED visits occurring among mostly female (56.8%) and middle-aged adults (mean [SD] age, 58.4 [16.3] years) and 1 911 795 diabetes-specific ED visits among adults with diabetes (eTable 5 in the Supplement). During the same period, for the 11 states included in our analysis, we identified 8 418 898 all-cause ED visits and 710 855 diabetes-specific ED visits among adults with diabetes.

### National Estimates

The national rate of all-cause diabetes ED visits (per 10 000 adults) increased from 257.6 (95% CI, 249.9-265.3) in 2008 to 400.8 (95% CI, 387.6-414.0) in 2016 and 2017, representing a 55.6% increase (95% CI, 50.6%-60.6%), outpacing the percentage increase in the annual rate of total ED visits (Table 1). The rate of diabetes-specific ED visits (per 10 000 adults) increased from 17.2 (95% CI, 16.7-17.7) in 2008 to 25.9 (95% CI, 25.1-26.8) in 2016 and 2017, representing a 50.6% increase (95% CI, 46.3%-54.9%), with the greatest increase in rates occurring between 2014 and 2016.

**Table 1.** Total, All-Cause Diabetes, and Diabetes-Specific Rates of ED Use per 10 000 Adults, 2008-2017

Visit type	2008		2011		2014		2016-2017^a^		Change over 2008-2017	
	No.	Rate (95% CI)^b^	No.	Rate (95% CI)^b^	No.	Rate (95% CI)^b^	No.	Rate (95% CI)^b^	Absolute	% (95% CI)
Total^c^										
National	80 261 409	3466.5 (3387.1-3545.8)	85 454 357	3589.0 (3512.9-3665.2)	92 979 514	3792.6 (3693.5-3891.7)	98 075 812	3935.6 (3826.1-4045.2)	469.1	13.5 (10.2-16.8)
11-State sample	19 791 913	3223.0 (3221.6-3224.4)	22 320 973	3542.4 (3540.9-3543.9)	24 513 826	3768.9 (3767.4-3770.4)	26 049 984	3879.8 (3878.3-3881.3)	656.8	20.4 (20.3-20.5)
All-cause diabetes^c^										
National	5 891 044	257.6 (249.9-265.3)	7 316 062	305.7 (297.0-314.3)	8 911 744	356.2 (344.3-368.1)	10 314 165	400.8 (387.6-414.0)	143.2	55.6 (50.6-60.6)
11-State sample	1 347 127	216.5 (216.2-216.9)	1 784 882	275.4 (275.0-275.8)	2 278 379	335.6 (335.2-336.1)	2 829 247	397.8 (397.3-398.2)	181.3	83.7 (83.5-83.9)
Diabetes-specific^c^										
National	394 818	17.2 (16.7-17.7)	415 170	17.4 (16.8-17.9)	447 276	18.0 (17.4-18.6)	654 531	25.9 (25.1-26.8)	8.7	50.6 (46.3-54.9)
11-State sample	91 296	14.7 (14.6-14.7)	98 978	15.3 (15.2-15.4)	109 234	16.3 (16.2-16.4)	179 534	26.1 (26.0-26.2)	11.4	78.1 (77.2-79.0)

Abbreviation: ED, emergency department.

^a^ National rates reflect 2016 estimates because 2017 data were not available. State mean rates reflect the mean of 2016 and 2017 rates.

^b^ Rates of ED use by state were calculated using numerator data from the Healthcare Cost and Utilization Project National Emergency Department Sample and State Emergency Department Databases and denominator data from the IPUMS USA American Community Survey from 2008 to 2016. All rates are age-standardized to the 2010 US adult population using population estimates available from the Centers for Disease Control and Prevention Mortality Database.

^c^ The total number of ED visits was 356 771 092, the total number of all-cause ED visits was 32 433 015, and the total number of diabetes-specific ED visits was 1 911 795. Total numbers reflect overall ED use (diabetes and nondiabetes visits).

All-cause diabetes ED visits increased across all groups studied, with percentage increases greatest among urban (58.3% [95% CI, 52.5%-64.1%]) and uninsured adults (75.3% [95% CI, 59.8%-90.8%]), as well as those living in the Northeast (69.6% [95% CI, 55.3%-83.9%]) and West (67.5% [95% CI, 56.5%-78.5%]) (Table 2). Rural patients had persistently higher rates of all-cause diabetes ED use compared with urban patients, as did Medicare and Medicaid patients compared with patients with other insurance types. Similarly, we also observed increased diabetes-specific ED use across all subgroups. Rates of diabetes-specific ED visits among urban patients from 2008 to 2016 increased by 54.4% (95% CI, 48.4%-60.4%), which was greater than the increase for rural patients (42.7% [95% CI, 36.4%-49.0%]). The greatest increase in percentage change in the rate of diabetes-specific ED visits was observed among uninsured patients (123.4% [95% CI, 103.9%-142.9%]).

**Table 2.** Age-Adjusted National Rates of All-Cause Diabetes and Diabetes-Specific ED Use per 10 000 Adults in the United States, 2008-2016

Variable	Rate per 10 000 adults (95% CI)^a^				Change over 2008-2016	
	2008	2011	2014	2016	Absolute	% (95% CI)
**All-cause diabetes visits^b^**						
Rural vs urban^c^						
Rural	310.7 (295.3-326.1)	352.6 (336.5-368.6)	384.3 (367.7-401.0)	457.0 (433.3-480.8)	146.3	47.1 (39.4-54.8)
Urban	246.3 (237.9-254.7)	295.9 (286.1-305.7)	350.9 (337.3-364.5)	389.9 (375.0-404.9)	143.6	58.3 (52.5-64.1)
Region^c^						
Midwest	292.0 (274.2-309.9)	360.1 (336.8-383.4)	417 (389.9-444.1)	457.9 (425.9-490.0)	165.9	56.8 (46.2-67.4)
Northeast	268.1 (247.3-288.8)	344.6 (319.6-369.6)	401.4 (367.5-435.2)	454.6 (414.3-495.0)	186.5	69.6 (55.3-83.9)
South	340.6 (325.2-356.0)	379.2 (364.2-394.2)	434.3 (409.7-458.9)	492.9 (467.2-518.5)	152.3	44.7 (37.3-52.1)
West	206.5 (192.0-221.0)	254.6 (237.0-272.3)	308.2 (288.8-327.5)	345.8 (323.0-368.5)	139.3	67.5 (56.5-78.5)
Insurance						
Medicaid	700.6 (653.0-748.1)	780.7 (728.4-833.1)	921.6 (852.0-991.1)	847.3 (782.2-912.4)	146.7	20.9 (11.2-30.6)
Medicare	601.8 (568.6-635.0)	712.8 (676.3-749.2)	803.9 (756.7-851.0)	896.8 (841.2-952.4)	295.0	49.0 (39.9-58.1)
Private	117.7 (110.2-125.3)	128.3 (120.7-135.8)	137.1 (127.2-147.1)	162.8 (151.8-173.9)	45.1	38.3 (28.8-47.8)
Uninsured	168.5 (154.5-182.4)	193.6 (175.3-212.0)	224.8 (199.0-250.6)	295.4 (267.7-323.1)	126.9	75.3 (59.8-90.8)
**Diabetes-specific visits^b^**						
Rural vs urban^c^						
Rural	22.7 (21.7-23.7)	21.7 (20.8-22.5)	22.5 (21.6-23.4)	32.4 (31.0-33.9)	9.7	42.7 (36.4-49.0)
Urban	16.0 (15.5-16.5)	16.5 (15.9-17.1)	17.1 (16.5-17.8)	24.7 (23.8-25.7)	8.7	54.4 (48.4-60.4)
Region^c^						
Midwest	18.4 (17.5-19.4)	19.3 (18.1-20.5)	21.1 (19.9-22.3)	29.2 (27.4-31.0)	10.8	58.7 (49.5-67.9)
Northeast	21.6 (20.3-23.0)	21.3 (19.7-22.8)	21.1 (19.3-22.9)	31.0 (28.5-33.6)	9.4	43.5 (32.2-54.8)
South	20.5 (19.6-21.4)	20.5 (19.6-21.4)	20.9 (19.7-22.0)	32.0 (30.3-33.7)	11.5	56.1 (47.8-64.4)
West	15.9 (14.9-17.0)	16.1 (14.9-17.2)	16.7 (15.6-17.8)	21.5 (20.1-23.0)	5.6	35.2 (25.6-44.8)
Insurance						
Medicaid	47.7 (44.5-50.8)	47 (43.7-50.2)	52.4 (48.3-56.5)	68.1 (62.9-73.2)	20.4	42.8 (32.2-53.4)
Medicare	40.1 (38.1-42.1)	38.4 (36.5-40.4)	37.2 (35.2-39.2)	45.4 (42.8-48.0)	5.3	13.2 (6.5-19.9)
Private	7.3 (6.9-7.8)	6.8 (6.3-7.2)	6.7 (6.2-7.1)	10.4 (9.7-11.0)	3.1	42.5 (34.4-50.6)
Uninsured	13.7 (12.6-14.8)	14 (12.5-15.6)	14.1 (12.4-15.7)	30.6 (27.5-33.6)	16.9	123.4 (103.9-142.9)

Abbreviation: ED, emergency department.

^a^ Rates of ED use by subgroup calculated using numerator data from the Healthcare Cost and Utilization Project National Emergency Department Sample and denominator data from the IPUMS USA American Community Survey from 2008 to 2016.

^b^ The total number of all-cause ED visits was 32 433 015, and the total number of diabetes-specific ED visits was 1 911 795.

^c^ Rural vs urban and region-specific rates are age-standardized to the 2010 US adult population using data from the Centers for Disease Control and Prevention Mortality Database.

In sensitivity analyses, we recalculated our rate of national all-cause diabetes ED visits using a denominator of people with diabetes and observed an increase in ED use (2016-2008 rate ratio, 1.45 [95% CI, 1.44-1.47]) relative to people without diabetes (2016-2008 rate ratio, 1.11 [95% CI, 1.10-1.12]) (eTable 6 in the Supplement). We observed evidence of additional diagnoses per diabetes-specific discharge (eTable 20 in the Supplement).

### State Estimates

**Figure 1.**
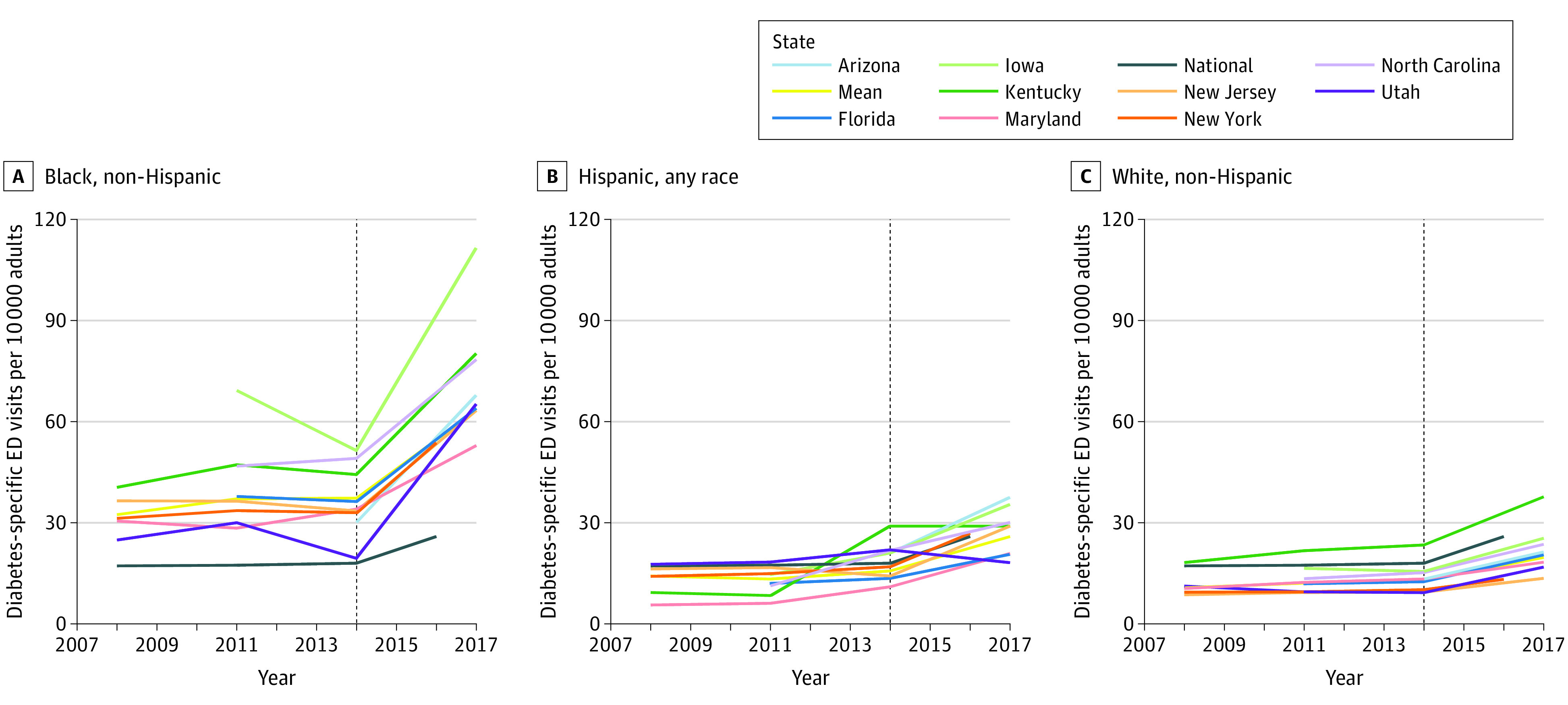
Age-Adjusted Rates of Diabetes-Specific Emergency Department (ED) Use Among US Adults by Race and Ethnicity, 2008-2017 Rates of ED use were calculated using numerator data from the Healthcare Cost and Utilization Project National Emergency Department Sample and State Emergency Department Databases and denominator data from the IPUMS USA American Community Survey from 2008 to 2017. All rates are age-standardized to the national 2010 US adult population using data from the Centers for Disease Control and Prevention Mortality Database. Two benchmark lines are plotted: the national line shows the national rate of ED use across all US adults, regardless of race and ethnicity, and the mean line shows mean rate of ED use across all states by race and ethnicity. Rates were not reported for state data with inconsistently coded race and ethnicity variables and/or subgroup estimates with estimates of 10 events or less or relative standard error of 30% or more. Nevada did not supply race and ethnicity data. The dotted vertical line delineates the point within our data sets at which the data transitioned from rates generated using *International Classification of Diseases, Ninth Revision, Clinical Modification* codes to rates generated using *International Statistical Classification of Diseases and Related Health Problems, Tenth Revision, Clinical Modification* codes. Rates after the transition may not be directly comparable to rates prior to the transition.

**Figure 2.**
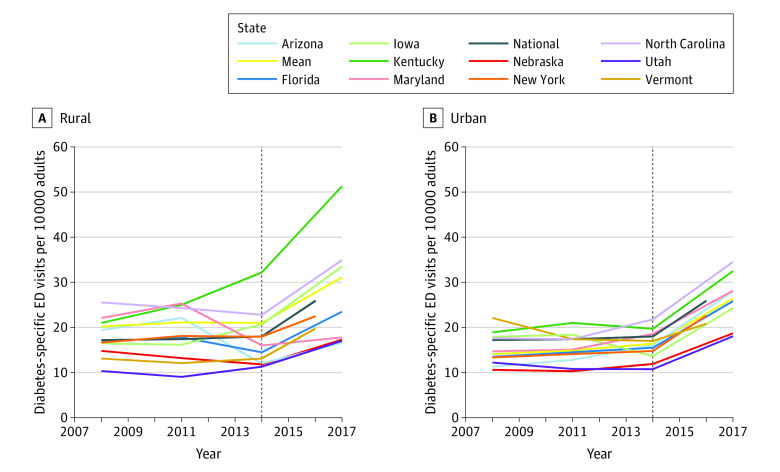
Age-Adjusted Rates of Diabetes-Specific Emergency Department (ED) Use Among US Adults by Rural or Urban Status, 2008-2017 Rates of ED use were calculated using numerator data from the Healthcare Cost and Utilization Project National Emergency Department Sample and State Emergency Department Databases and denominator data from the IPUMS USA American Community Survey from 2008 to 2017. All rates are age-standardized to the national 2010 US adult population using data from the Centers for Disease Control and Prevention Mortality Database. Two benchmark lines are plotted: the national line shows the national rate of ED use across all US adults, regardless of rural or urban status, and the mean line shows mean rate of ED use across all states by rural or urban status. Rates were not reported for state data with inconsistently coded race and ethnicity variables and/or subgroup estimates with 10 events or less or relative standard error more than 30%. The denominator of the rural-urban variable did not allocate any rural counties in New Jersey. The dotted vertical line delineates the point within our data sets at which the data transitioned from rates generated using *International Classification of Diseases, Ninth Revision, Clinical Modification* codes to rates generated using *International Statistical Classification of Diseases and Related Health Problems, Tenth Revision, Clinical Modification* codes. Rates after the transition may not be directly comparable to rates prior to the transition.

**Figure 3.**
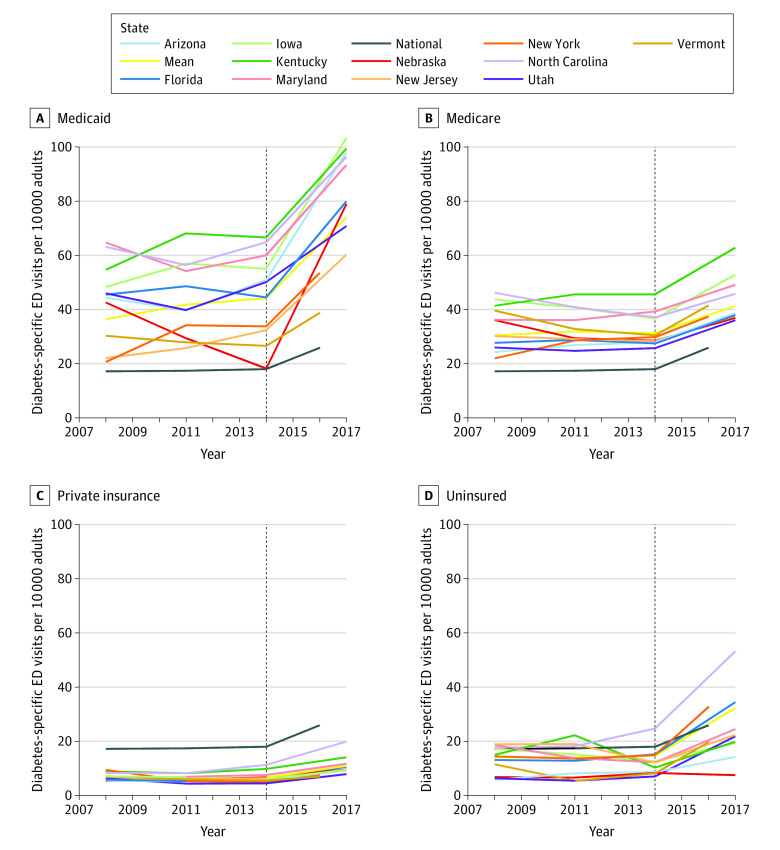
Insurance-Specific Rates of Diabetes-Specific Emergency Department (ED) Use Among US Adults, 2008-2017 Rates of ED use were calculated using numerator data from the Healthcare Cost and Utilization Project National Emergency Department Sample and State Emergency Department Databases and denominator data from the IPUMS USA American Community Survey from 2008 to 2017. Two benchmark lines are plotted: the national line shows the national rate of ED use across all US adults, regardless of insurance status, and the mean line shows the mean rate of ED use across all states by insurance group. The dotted vertical line delineates the point within our data sets at which the data transitioned from rates generated using *International Classification of Diseases, Ninth Revision, Clinical Modification* codes to rates generated using *International Statistical Classification of Diseases and Related Health Problems, Tenth Revision, Clinical Modification* codes. Rates after the transition may not be directly comparable to rates prior to the transition.

The total rates of all-cause diabetes and diabetes-specific ED visits from our 11-state sample were slightly lower than those generated from the national data, while trends were similar during the study period (Table 1). Although we focused on diabetes-specific ED use in Figure 1, Figure 2, and Figure 3 (values in eTables 8-19 in the Supplement), we also examine the variation in all-cause diabetes ED use and found a stronger upward trend across groups (eFigures 3, 4, and 5 in the Supplement).

On average across states and years, Black adults had roughly 3 times (rate ratio, 3.09 [95% CI, 2.91-3.30]) the rate of diabetes-specific ED use compared with White adults, with no significant increase or decrease during the study period (Figure 1 values in eTable 7 in the Supplement). On average, Hispanic adults had a 29% higher rate of ED visits (rate ratio, 1.29 [95% CI, 1.19-1.40]) compared with White adults; similarly, there was no significant narrowing or widening of disparate ED use during the study period. Except for Kentucky (rate in 2017, 37.7 [95% CI, 37.0-38.4]), White adults in each state in our sample across all years had lower rates of diabetes-specific ED visits compared with the national average for all races and ethnicities (rate in 2016, 25.9 [95% CI, 25.1-26.8]) (Table 1).

Although the rate of ED use among Black adults with diabetes was generally higher than that for White adults from the same state, there was notable heterogeneity in the rate of ED use across states. We observed a low rate of ED use among Black adults in Maryland (rate in 2008, 30.5 [95% CI, 29.5-31.6]; rate in 2017, 52.9 [95% CI, 51.6-54.1]) (eTable 12 in the Supplement) and the highest rate of ED use among Black adults in Iowa (rate in 2011, 69.2 [95% CI, 60.7-77.7]; rate in 2017, 111.5 [95% CI, 102.9-120.1]) (eTable 10 in the Supplement). Across states, race-specific trends were less variable for White adults; for instance, we observed low rates for White adults in New York (rate in 2008, 9.4 [95% CI, 9.2-9.6]; rate in 2017, 13.2 [95% CI, 13.0-13.4]) (eTable 16 in the Supplement) and the highest rate among White adults in Kentucky (rate in 2008, 18.2 [95% CI, 17.7-18.7]; rate in 2017, 37.7 [95% CI, 37.0-38.4]) (eTable 11 in the Supplement).

Collectively, the rates of diabetes-specific ED visits across states and years for the rural group were 34% higher (rate ratio, 1.34 [95% CI, 1.26-1.44]) than the rates for the urban group (Figure 2; eTable 7 in the Supplement). There was no uniform pattern observed in the rates of ED visits in rural and urban areas across states; while we observed decreasing rates of ED use among rural patients from 2008 to 2014 in some states (Florida, North Carolina, and Maryland), others experienced increased rates of ED use among rural patients (New York, Utah, Kentucky) (eTables 9, 11, 12, 13, 16, and 17 in the Supplement). However, there was greater variation in state-specific trends in ED use for rural adult populations than for urban adult populations. Kentucky experienced a sizable increase in the rate of ED use among rural patients since 2011 and has the highest rate of ED use among rural patients as of 2017 (rate in 2011, 25.0 [95% CI, 24.2-25.8]; rate in 2017, 51.3 [95% CI, 50.1-52.5]) (eTable 11 in the Supplement). During this period, Kentucky also became the state with the widest rural vs urban difference (urban rate in 2011, 21.0 [95% CI, 20.4-21.7]; urban rate in 2017, 32.5 [95% CI, 31.7-33.3]). Meanwhile, rates of ED use among rural patients in Arizona peaked in 2011 (rate in 2011, 22.1 [95% CI, 20.8-23.4]), and the state has the lowest rate of ED use among rural patients as of 2017 (rate in 2017, 16.6 [95% CI, 15.5-17.8]) (eTable 8 in the Supplement).

The mean rates of ED use across states and years were much higher among Medicaid (rate ratio, 6.65 [95% CI, 6.49-6.82]) and Medicare patients (rate ratio, 4.37 [95% CI, 4.23-4.51]) compared with privately insured adults (eTable 7 in the Supplement). The rate of diabetes-specific ED use among uninsured patients was more than twice (rate ratio, 2.25 [95% CI, 2.12-2.40]) that among privately insured adults. On average across states, we observed that Medicaid beneficiaries had the largest increase in the rate of diabetes-specific ED use per 10 000 visits, from 36.4 in 2008 (95% CI, 35.7-37.0) to 44.2 in 2014 (95% CI, 43.7-44.8) (Figure 3 and eTable 19 in the Supplement). Consistently across years, Medicaid beneficiaries and uninsured adults had greater variability in ED use across states compared with more consistent ED use among patients with private insurance and those with Medicare (Figure 3). We observed high rates of ED use among patients with Medicaid in Iowa (rate in 2008, 48.3 [95% CI, 44.0-52.7]; rate in 2017, 103.4 [95% CI, 99.0-107.9]) (eTable 10 in the Supplement) and the lowest rates of ED use among patients with Medicaid in Vermont (rate in 2008, 30.3 [95% CI, 25.3-35.4]; rate in 2017, 38.8 [95% CI, 34.2-43.5]) (eTable 18 in the Supplement). The rates of diabetes-specific visits were highest among patients with Medicaid in Kentucky, North Carolina, Iowa, and Arizona, while the rates for uninsured patients remained below the total national mean rates for all insurance groups from 2008 to 2017 in all cases except for North Carolina, Maryland, Florida, and New York (eTables 8, 9, 10, 11, 13, and 16 in the Supplement).

## Discussion

This study offers new empirical evidence of marked disparities, both nationally and within states, in age-adjusted, all-cause, and diabetes-specific “treat-and-release” ED use by race and ethnicity, region, rural vs urban location, and insurance type from 2008 to 2017. We used high-quality administrative discharge databases that have previously been used only to describe aggregate ED use with cross-sectional, national estimates.^2,27^ All-cause diabetes treat-and-release ED use among individuals with diabetes increased markedly during the study period, outpacing the increase in rates of overall treat-and-release ED use, while the rates of diabetes-specific ED use were relatively stable, with a slight increase. We found persistently higher rates of diabetes-specific treat-and-release ED use among rural, Black, and Medicare- and Medicaid-insured adults, with large state-to-state variations within each group that were not reflected in national trends. The persistence of these disparities calls for focused local and federal efforts to understand and address the underlying reasons for differential ED use.

Because previous estimates of diabetes-related ED use in the US examined use for specific diabetes complications, relied on data that underreport diabetes-related conditions, or did not examine state-level use and within-state use (ie, rural vs urban stratification by state),^25,26,44,45,46^ direct comparisons of our estimates are not possible. The prevalence of diagnosed diabetes has increased from 7.9% in 2008 to 8.5% in 2017,^47^ which may be associated with increases in ED use, although national all-cause ED use among people with diabetes outpaced all-cause ED use among those without diabetes when controlling for increased prevalence. Increases in the rates of all-cause diabetes ED use may also reflect recently introduced incentives to capture comorbid conditions and to reduce inpatient admissions.^47,48^ Furthermore, reported decreases in the rates of diabetes-related inpatient admissions in the context of increasing rates of all-cause treat-and-release ED visits may reflect increasing case acuity and use of ED services to manage this patient population.^48,49^

The rates of diabetes-specific ED visits were markedly and persistently different by race and ethnicity. Black individuals visited the ED 3 times more than White individuals and approximately 2 times more than Hispanic individuals from 2008 to 2017, consistent with previous findings on racial disparities in diabetes-related ED use.^21,44,50^ These racial and ethnic disparities persisted despite insurance coverage gains from the ACA and efforts by payers and health systems (eg, via care coordination) to lower rates of hospital and ED use. Although between-state variations were evident, rates over time for Kentucky, the state with the highest rate of diabetes-specific ED use among White patients, remained below the lowest rates for the Black population over time, which were found in Maryland. Research using self-reported data demonstrated similar aggregate disparities among racial and ethnic minority populations,^50^ which are consistent with our findings.

On average across years, the rates of diabetes-specific ED use among rural patients were 34% higher compared with the rates of diabetes-specific ED use among urban patients, with significant variations between states. Kentucky was one of the few Southern states to expand Medicaid coverage at the time of ACA implementation; however, it remains an outlier of higher ED use compared with other states and in terms of rural vs urban differences in ED use. Nationally, we observed greater percentage increases in diabetes-related ED use among urban patients compared with rural patients, although the rates of ED use among rural patients consistently remained higher than the rates of ED use among urban patients. Our estimates may reflect a state-specific lack of access to primary care in rural communities despite coverage gains. Previous examination found that low-income rural residents in Medicaid expansion states experienced greater coverage uptake compared with urban residents, although only urban residents reported greater access to care and reduced cost burdens.^18^ Furthermore, rural hospital closures accelerated in the period studied, particularly in Kentucky and North Carolina, reducing access to care in rural settings.^18,51,52^ These differential factors may be associated with the observed increased aggregate rates of ED use among urban patients as well as the state-specific variations in the rates of ED use among rural patients.

The period studied included major health reform efforts, such as the ACA, which expanded health insurance coverage to more than 20 million people in the US. The ACA is of great importance in population health for individuals with diabetes because uninsured persons tend to have worse diabetes control and are less likely to achieve the recommended processes of diabetes care.^53,54^ Although we did not explicitly test for the association of the implementation of the ACA with the rates of ED, we did not observe any visually discernible evidence of an association between rates of ED use and policy nationally or by state in our analysis of rates by payer. Eligibility expansions, growing inequality, and the lingering effects of the Great Recession are also reflected in our reported trends.^55,56^ The rates of ED use were consistently highest among adults insured by Medicaid or Medicare. This finding is expected given that individuals with high rates of ED use tend to be insured by Medicare or Medicaid.^57^ Although Medicaid was originally intended to support people experiencing hardship, it now covers 18% of the US population and 24% of those with diabetes.^58^ Because Medicaid and Medicare are taxpayer funded, costly ED use that is potentially preventable through improved outpatient and community interventions for diabetes should be a focus for future health reform.

We found wider state-to-state variations in the rates of ED visits among Black, Medicaid, and rural populations. Each of these groups has been documented to have comparatively less access to care than, respectively, White, privately insured, and urban populations.^21,22,51^ These findings emphasize that national trends do not adequately reflect the between-state and within-state variations and highlight the importance of examining trends in narrower geographic areas to identify high-risk states and subgroups. Consequential policy action must be taken to expand access to historically marginalized and disadvantaged populations; otherwise the observed disparities will remain unchanged or worsen.

### Limitations

This study has some limitations, including its reliance on administrative hospital discharge data. Clinicians, hospitals, and states may each vary in how accurately and comprehensively they code, which may result in coding inconsistencies between discharge records.^59^ In addition, underlying temporal changes in coding practices may have affected the reported trends, such as the adoption of value-based payment systems, which may have incentivized clinicians to code for more comorbid conditions. Our analysis of diabetes-specific ED visits circumvented temporal coding changes for additional diagnoses because we examined only complications based on the principal diagnosis. In addition, because the quality of the data on the race and ethnicity variable varied between states, we did not report race- and ethnicity-specific estimates for state-years in which coding was not consistent with the uniform variable or for which data on race and ethnicity were not available. We were also limited in our analysis to states that consistently provided data to HCUP during the period studied. This limitation is offset somewhat by the large volume of data and the demographic and geographic diversity of the state populations covered.^60,61^ Furthermore, because our unit of observation was ED discharge records rather than people with diabetes, we are unable to identify the proportion of ED use by patients with a high rate of ED use. As a result, patients who use ED services more frequently may be overrepresented in our sample. Diabetes complications, such as diabetic ketoacidosis, that primarily result in admissions to the ED are largely not represented in these trends because we examined treat-and-release ED visits.^49^ Furthermore, as this was a trend analysis encompassing 2008 to 2017, the 2015 transition between the use of *ICD-9-CM* codes and the use of *ICD-10-CM* codes to identify diabetes-related ED visits may have introduced potential volatility in trends. Although we selected comparable *ICD-9* and *ICD-10* codes, the trends before and after the transition may be the result of actual changes in rates or the result of the transition.

## Conclusions

This cross-sectional study reported on the national and state-level estimates generated by a large administrative discharge record database of ED visits not resulting in hospitalization in the US. In general, ED use by adults with diabetes increased over time. National trends were not always reflective of the between- and within-state heterogeneity in trends in ED use shown in our analyses. We noted wide variations in ED use by racial and ethnic subgroups, rural and urban subgroups, and insurance subgroups. These disparities persisted from 2008 to 2017, despite passage and implementation of major health care reforms. Future policy research and implementation to reduce the burden of diabetes should go beyond coverage gains and delve more into the social determinants of health and equity specific to state-level and substate-level regions.

## Notes

Supplement. **eFigure 1.** NEDS Data Selection **eFigure 2.** SEDD Data Selection **eTable 1.** Missing Observation and Percentage Missing by Variable for NEDS Dataset **eTable 2.** Descriptive Statistics of NEDS Discharge Records With Complete Data Compared to Data With Missing Observations for ≥1 Analytic Variable **eTable 3.** Missing Observations and Percentage Missing by Variable for SEDD Datasets With >1% Missingness for Analytic Variables **eAppendix 1.** Study Selection and Missing Data Methods **eTable 4.** *ICD-9* and *ICD-10* Code List to Define Diabetes-Specific Events **eAppendix 2.** eMethods Calculating Crude Rates, Age-Standardized Rates, and Standardized Rate Ratios **eTable 5.** Sample Characteristics of All-Cause and Diabetes-Specific ED Visits From the National Emergency Department Dataset, Combined Data From 2008, 2011, 2014, 2016 **eAppendix 3.** Analysis by Diabetes Status **eTable 6.** Age Adjusted Rates of Average Annual Recorded Emergency Department Visits (per 1000 Adults) Among US Adult Population With and Without Diabetes From 2008-2016 **eTable 7.** Rate Ratio of Average Annual Diabetes-Specific Emergency Department Visits (per 10,000 Adults) Among US Adult Population From 2008-2017 **eTable 8.** Rates of Diabetes-Specific Emergency Department Visits per 10,000 Adults in Arizona; 2008-2017 **eTable 9.** Rates of Diabetes-Specific Emergency Department Visits per 10,000 Adults in Florida; 2008-2017 **eTable 10.** Rates of Diabetes-Specific Emergency Department Visits per 10,000 Adults in Iowa; 2008-2017 **eTable 11.** Rates of Diabetes-Specific Emergency Department Visits per 10,000 Adults in Kentucky; 2008-2017 **eTable 12.** Rates of Diabetes-Specific Emergency Department Visits per 10,000 Adults in Maryland; 2008-2017 **eTable 13.** Rates of Diabetes-Specific Emergency Department Visits per 10,000 Adults in North Carolina; 2008-2017 **eTable 14.** Rates of Diabetes-Specific Emergency Department Visits per 10,000 Adults in Nebraska; 2008-2017 **eTable 15.** Rates of Diabetes-Specific Emergency Department Visits per 10,000 Adults in New Jersey; 2008-2017 **eTable 16.** Rates of Diabetes-Specific Emergency Department Visits per 10,000 Adults in New York; 2008-2016 **eTable 17.** Rates of Diabetes-Specific Emergency Department Visits per 10,000 Adults in Utah; 2008-2016 **eTable 18.** Rates of Diabetes-Specific Emergency Department Visits per 10,000 Adults in Vermont; 2008-2017 **eTable 19.** Average Rates of Diabetes-Specific Emergency Department Visits From Pooled State Data by Race, Rural/Urban, and Insurance; 2008-2017 **eFigure 3.** Age-Adjusted Rates of All-Cause Diabetes Emergency Department Use Among US Adults by Race/Ethnicity; 2008-2017 **eFigure 4.** Age-Adjusted Rates of All-Cause Diabetes Emergency Department Use Among US Adults by Rural Status; 2008-2017 **eFigure 5.** Insurance-Specific Rates of Diabetes-Specific Emergency Department Use Among US Adults; 2008-2017 **eTable 20.** Proportion of Diabetes-Specific Emergency Department Discharges by Number of Diagnoses on Record; 2008-2016
eReferences.
