# Supplementary material for: Trends and Variations in Emergency Department Use Associated With Diabetes in the US by Sociodemographic Factors, 2008-2017
Source: JAMA Netw Open. 2022 May 25;5(5):e2213867. doi: 10.1001/jamanetworkopen.2022.13867 (PMC9133946; doi:10.1001/jamanetworkopen.2022.13867)
Supplement: Supplement. — eFigure 1. NEDS Data Selection eFigure 2. SEDD Data Selection eTable 1. Missing Observation and Percentage Missing by Variable for NEDS Dataset eTable 2. Descriptive Statistics of NEDS Discharge Records With Complete Data Compared to Data With Missing Observations for ≥1 Analytic Variable eTable 3. Missing Observations and Percentage Missing by Variable for SEDD Datasets With >1% Missingness for Analytic Variables eAppendix 1. Study Selection and Missing Data Methods eTable 4. ICD-9 and ICD-10 Code List to Define Diabetes-Specific Events eAppendix 2. eMethods Calculating Crude Rates, Age-Standardized Rates, and Standardized Rate Ratios eTable 5. Sample Characteristics of All-Cause and Diabetes-Specific ED Visits From the National Emergency Department Dataset, Combined Data From 2008, 2011, 2014, 2016 eAppendix 3. Analysis by Diabetes Status eTable 6. Age Adjusted Rates of Average Annual Recorded Emergency Department Visits (per 1000 Adults) Among US Adult Population With and Without Diabetes From 2008-2016 eTable 7. Rate Ratio of Average Annual Diabetes-Specific Emergency Department Visits (per 10,000 Adults) Among US Adult Population From 2008-2017 eTable 8. Rates of Diabetes-Specific Emergency Department Visits per 10,000 Adults in Arizona; 2008-2017 eTable 9. Rates of Diabetes-Specific Emergency Department Visits per 10,000 Adults in Florida; 2008-2017 eTable 10. Rates of Diabetes-Specific Emergency Department Visits per 10,000 Adults in Iowa; 2008-2017 eTable 11. Rates of Diabetes-Specific Emergency Department Visits per 10,000 Adults in Kentucky; 2008-2017 eTable 12. Rates of Diabetes-Specific Emergency Department Visits per 10,000 Adults in Maryland; 2008-2017 eTable 13. Rates of Diabetes-Specific Emergency Department Visits per 10,000 Adults in North Carolina; 2008-2017 eTable 14. Rates of Diabetes-Specific Emergency Department Visits per 10,000 Adults in Nebraska; 2008-2017 eTable 15. Rates of Diabetes-Specific Emergency Department Visits per 10,000 Adults i [file jamanetwopen-e2213867-s001.pdf]

Supplementary Online Content

Uppal TS, Chehal PK, Fernandes G, et al. Trends and variations in emergency department use associated with diabetes in the US by sociodemographic factors, 2008-2017. *JAMA Netw Open.* 2022;5(5):e2213867. doi:10.1001/jamanetworkopen.2022.13867

eFigure 1. NEDS Data Selection
eFigure 2. SEDD Data Selection
eTable 1. Missing Observation and Percentage Missing by Variable for NEDS Dataset
eTable 2. Descriptive Statistics of NEDS Discharge Records With Complete Data Compared to Data With Missing Observations for ≥1 Analytic Variable
eTable 3. Missing Observations and Percentage Missing by Variable for SEDD Datasets With >1% Missingness for Analytic Variables
eAppendix 1. Study Selection and Missing Data Methods
eTable 4. *ICD-9* and *ICD-10* Code List to Define Diabetes-Specific Events
eAppendix 2. eMethods Calculating Crude Rates, Age-Standardized Rates, and Standardized Rate Ratios
eTable 5. Sample Characteristics of All-Cause and Diabetes-Specific ED Visits From the National Emergency Department Dataset, Combined Data From 2008, 2011, 2014, 2016
eAppendix 3. Analysis by Diabetes Status
eTable 6. Age Adjusted Rates of Average Annual Recorded Emergency Department Visits (per 1000 Adults) Among US Adult Population With and Without Diabetes From 2008-2016
eTable 7. Rate Ratio of Average Annual Diabetes-Specific Emergency Department Visits (per 10,000 Adults) Among US Adult Population From 2008-2017
eTable 8. Rates of Diabetes-Specific Emergency Department Visits per 10,000 Adults in Arizona; 2008-2017
eTable 9. Rates of Diabetes-Specific Emergency Department Visits per 10,000 Adults in Florida; 2008-2017
eTable 10. Rates of Diabetes-Specific Emergency Department Visits per 10,000 Adults in Iowa; 2008-2017
eTable 11. Rates of Diabetes-Specific Emergency Department Visits per 10,000 Adults in Kentucky; 2008-2017
eTable 12. Rates of Diabetes-Specific Emergency Department Visits per 10,000 Adults in Maryland; 2008-2017
eTable 13. Rates of Diabetes-Specific Emergency Department Visits per 10,000 Adults in North Carolina; 2008-2017
eTable 14. Rates of Diabetes-Specific Emergency Department Visits per 10,000 Adults in Nebraska; 2008-2017
eTable 15. Rates of Diabetes-Specific Emergency Department Visits per 10,000 Adults in New Jersey; 2008-2017
eTable 16. Rates of Diabetes-Specific Emergency Department Visits per 10,000 Adults in New York; 2008-2016
eTable 17. Rates of Diabetes-Specific Emergency Department Visits per 10,000 Adults in Utah; 2008-2016
eTable 18. Rates of Diabetes-Specific Emergency Department Visits per 10,000 Adults in Vermont; 2008-2017
eTable 19. Average Rates of Diabetes-Specific Emergency Department Visits From Pooled State Data by Race, Rural/Urban, and Insurance; 2008-2017
eFigure 3. Age-Adjusted Rates of All-Cause Diabetes Emergency Department Use Among US Adults by Race/Ethnicity; 2008-2017
eFigure 4. Age-Adjusted Rates of All-Cause Diabetes Emergency Department Use Among US Adults by Rural Status; 2008-2017
eFigure 5. Insurance-Specific Rates of Diabetes-Specific Emergency Department Use Among US Adults; 2008-2017
eTable 20. Proportion of Diabetes-Specific Emergency Department Discharges by Number of Diagnoses on Record; 2008-2016
eReferences.

This supplementary material has been provided by the authors to give readers additional information about their work.

© 2022 Uppal TS et al. *JAMA Network Open.*

eFigure 1. NEDS Data Selection

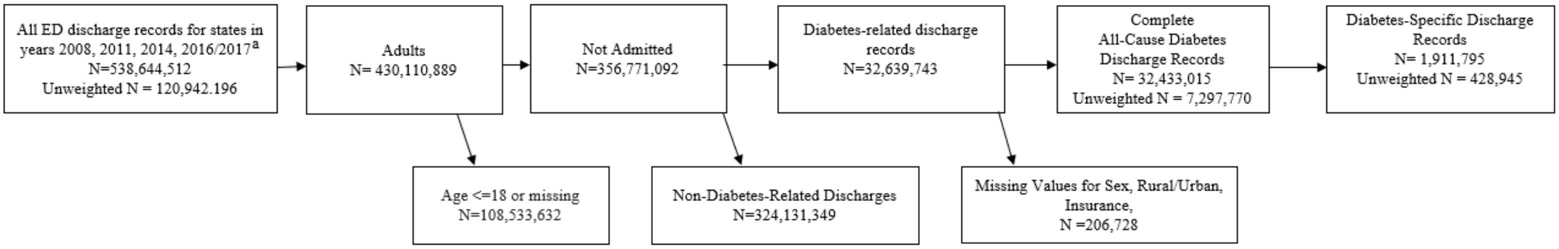

© 2022 Uppal TS et al. *JAMA Network Open.*

eFigure 2. SEDD Data Selection

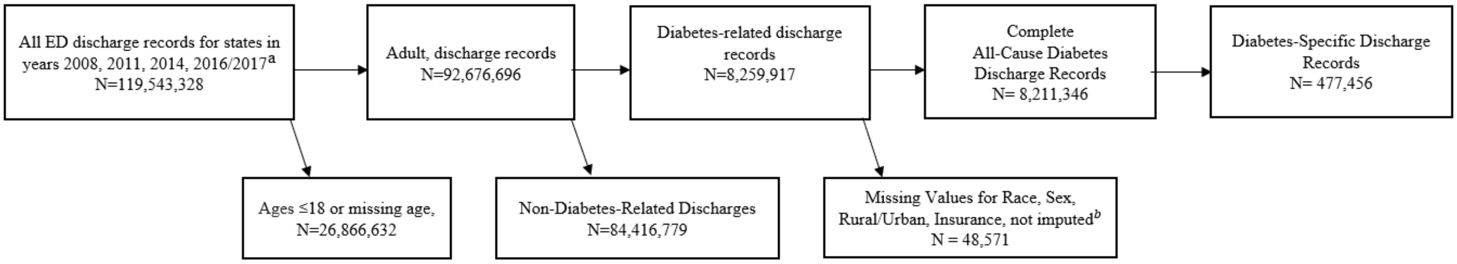

© 2022 Uppal TS et al. *JAMA Network Open.*

eTable 1. Missing Observation and Percentage Missing by Variable for NEDS Dataset

Variable Name	Variable Type	Total Number of Missing Observations (%) - NEDS				Description
		2008	2011	2014	2016	
Gender	Binary	482 (0.02)	455 (0.02)	84 (0.00)	856 (0.02)	Male/Female
Patient Location - NCHS categories	Ordered Categorical	15,432 (0.62)	9,779 (0.35)	15,549 (0.47)	14,880 (0.41)	1."Central" Metro counties ≥ 1 million population<br>2. "Fringe" Metro counties ≥1 million population<br>3. Metro counties 250,000-999,999 population<br>4. Metro counties 50,000-249,999 population<br>5. Micropolitan counties<br>6. Not metropolitan or microplitan counties
Primary Payer	Unordered Categorical	6002 (0.24)	5566 (0.20)	3200 (0.10)	2479 (0.07)	1. Medicare<br>2. Medicaid<br>3. Private insurance<br>4. Self-pay<br>5. No charge<br>6. Other
Total # of Observations (Unweighted)		2,473,676	2,764,598	3,281,887	3,625,092	

Within the NEDS datasets, we observed missingness primarily in patient location (rural/urban), and the primary payer variables. Our year specific NEDS datasets did not have missingness >1% for any of our analytical variables (**eTable 1)**. Analyses conducted using NEDS data were conducted using complete case selection. 0.63% of observations within the combined NEDS datasets had any missing value for analytical variables and were excluded for complete case analysis.

© 2022 Uppal TS et al. *JAMA Network Open.*

eTable 2. Descriptive Statistics of NEDS Discharge Records With Complete Data Compared to Data With Missing Observations for ≥1 Analytic Variable

	Complete All-Cause Diabetes ED Visits		Data with Missing Observation for ≥1 variable	
# of Visits				
Weighted		32,433,015		206,728
s.e.		(658,144)		(14,982)
Age (Mean)		58.4		54.6
s.e.		(0.08)		(0.40)
Age Groups				
18-29	4.6	(4.5, 4.7)	5.7	(5.1, 6.3)
30-44	15.9	(15.7, 16.1)	18.4	(17.2, 19.6)
45-64	42.6	(42.3, 42.8)	51.4	(49.9, 52.9)
65-74	17.1	(17, 17.3)	13.4	(12.5, 14.4)
75+	19.9	(19.5, 20.2)	11.1	(9.6, 12.6)
% Female	56.8	(56.5, 57)	45.4	(43.7, 47.1)
% Rural	20.0	(18.9, 21)	29.9	(21.3, 38.5)
Region				
Midwest	22.9	(21.3, 24.5)	17.0	(10.2, 23.8)
Northeast	14.5	(13, 15.9)	25.1	(18.5, 31.7)
South	43.5	(41.5, 45.5)	36.1	(29.4, 42.8)
West	19.2	(17.8 20.6)	21.8	(17.7, 25.9)
Insurance				
Medicaid	18.3	(17.8, 18.8)	24.7	(21.5, 27.8)
Medicare	46.6	(46.1, 47.1)	26.7	(24.1, 29.4)
Private	22.6	(22.1, 23.1)	18.9	(16.7, 21)
Uninsured	9.1	(8.6, 9.5)	23.0	(19.5, 26.6)
Other	3.4	(3.2, 3.7)	6.7	(4.1, 9.4)

Dataset generated from National Emergency Department Sample data, years 2008, 2011, 2014, 2016. All-cause diabetes visits include all discharge records with a diabetes-related diagnosis, while diabetes-specific visits include all visits with a prinicpal diagnosis of a diabetes specific condition/complication.

Discharge records within the NEDS dataset with missing values were more likely to be from patients that were rural (missing: 29.9%; complete: 20.0%), Medicaid-insured (missing: 24.7%; complete: 18.3%), or Uninsured (missing: 23.0%; complete: 9.1%) (**eTable 2.)**

© 2022 Uppal TS et al. *JAMA Network Open.*

eTable 3. Missing Observations and Percentage Missing by Variable for SEDD Datasets With >1% Missingness for Analytic Variables

Variable Name	Variable Type	Total Number of Missing Observations (%) - SEDD													Description
		Florida (2017)	Iowa (2008)	Iowa (2011)	Iowa (2014)	Maryland (2014)	North Carolina (2008)	New York (2008)	New York (2014)	New York (2016)	Utah (2008)	Utah (2011)	Utah (2014)	Utah (2017)	
Gender	Binary	1 (0.00)	2 (0.00)	1 (0.00)	2 (0.00)	-	5 (0.00)	3 (0.00)	6 (0.00)	6 (0.00)	1 (0.00)	4 (0.01)	-	2 (0.00)	Male/female
Race	Unordered categorical	2514 (0.35)	3232 (6.98)*	2348 (4.34)*	1636 (2.97)*	24023 (13.65)*	96216 (40.23)*	1718 (0.79)	-	-	2189 (6.58)*	1865 (5.23)*	3046 (7.64)*	2530 (5.60)*	1. White<br>2. Black<br>3. Hispanic<br>4. Asian or Pacific Islander<br>5. Native American<br>6. Mixed-race
Patient Location - NCHS categories	Ordered Categorical	7605 (1.05)*	47 (0.10)	18 (0.03)	5 (0.01)	425 (0.24)	1446 (0.60)	6099 (2.83)*	4992 (1.13)*	6173 (1.21)*	66 (0.20)	65 (0.18)	63 (0.16)	74 (0.16)	1."Central" Metro counties ≥ 1 million population<br>2. "Fringe" Metro counties ≥1 million population<br>3. Metro counties 250,000-999,999 population<br>4. Metro counties 50,000-249,999 population<br>5. Micropolitan counties<br>6. Not metropolitan or microplitan counties
Primary Payer	Unordered Categorical	36 (0.00)	-	120 (0.22)	-	19 (0.01)	731 (0.31)	-	114 (0.03)	139 (0.03)	493 (1.48)*	674 (1.89)*	684 (1.71)*	821 (1.82)*	1. Medicare<br>2. Medicaid<br>3. Private insurance<br>4. Self-pay<br>5. No charge<br>6. Other
Total # of Observations		727,053	46,282	54,132	55,145	175,934	239,156	215,414	441,361	508,245	33,278	35,689	39,901	45,213	

*Variable missing in >1% of total # of observations for given state-year dataset
"-" indicates no missing observations for the given dataset-variable

© 2022 Uppal TS et al. *JAMA Network Open.*

eAppendix 1. Study Selection and Missing Data Methods

Within the SEDD datasets, we observed missingness primarily in race, patient location (rural/urban), and the primary payer variables Within the SEDD datasets, missingness for race for the imputed state-year datasets ranged from 0.03% to 40.23%, as observed in North Carolina. Race-specific estimates for North Carolina in 2008 were not reported because of data quality issues reported by HCUP. The missingness for the patient location variable remained below 3% in all datasets, while primary payer missingness remained below 2%. 0.59% of the observations within the combined SEDD datasets had data missing for analytical variables (**eTable 3**).

To impute these missing observations, we used an algorithm proposed and evaluated by Ma, Zhang, Lyman, Huang [1,2]. Ma et al., focus explicitly on imputing missing data in the HCUP SID making it an ideal approach for our work. They show that conditional multiple imputation (MI) is the optimal approach when using HCUP data. We applied their algorithm to impute data for variables missing greater than 1% per year. The algorithm was applied to each state by year dataset. We did not impute value for variables that were completely missing each year and state. Consequently, states such as NE which did not collect race data were not imputed and excluded from our state-specific race estimates. Further, states such as NC and IA that had race data which was not uniformly coded throughout years were imputed however, race-specific rates were not reported in our state specific race estimates.

As noted by Ma et al., it is impossible to directly test whether the data are missing not at random (MNAR). For practical purposes we similarly assume that the data are missing at random (MAR) which has been recommended for practical applications especially when there are a large number of predictors of that can be included in the imputation model.[3-5] the HCUP data itself includes many quality predictors but following MA et al., we additionally include information on racial and socioeconomic status distributions from the U.S. Census and information on hospital characteristics the American Hospital Association database.

Conditional MI imputes data variable by variable rather than using a joint distribution. The benefit of this approach that it allows different types of variables to be modeled separately (e.g., categorical versus continuous variables). We use the MICE algorithm in R.[6] We evaluated imputation performance using a Markov chain Monte Carlo method which can evaluate whether the chain converges to a stationary distribution. Rates were generated from imputed datasets by estimating counts of visits in each imputed dataset separately and were combined using Rubin's rules.[7]

© 2022 Uppal TS et al. *JAMA Network Open.*

eTable 4. *ICD-9* and *ICD-10* Code List to Define Diabetes-Specific Events

	ICD-9-CM/PCS	*ICD-10-CM*/PCS
Short-Term Complications[8]	250.10, 250.11, 250.12, 250.13, 250.20, 250.21, 250.22, 250.23, 250.30, 250.31, 250.32, 250.33	E10.10, E10.11, E10.65, E10.69, E11.00, E11.01, E11.10, E11.641, E11.65, E11.69, E13.00, E13.01, E13.10, E13.641, E13.65, E13.69
Long-Term Complications[8]	250.4, 250.40, 250.41, 250.42, 250.43, 250.5, 250.51, 250.52, 250.53, 250.6, 250.61, 250.62, 250.63, 250.7, 250.70, 250.71, 250.72, 250.73, 250.8, 250.80, 250.81, 250.82, 250.83, 250.9, 250.90, 250.91, 250.92, 250.93	E1021, E1029, E10311, E10319, E1036, E1039, E1040, E1051, E1052, E1059, E10618, E10620, E10621, E10622, E10628, E10630, E10638, E10649 E1065, E1069, E108, E118, E1121, E1129, E11311, E1136, E11319, E1139, E1140, E1151, E1152, E1159, E11618, E11620, E11621, E11622, E11628, E11630, E11638, E11649, E1165, E1169, E1321, E1322, E1329, E13311, E13319, E1336, E1339, E1340, E1351, E1352, E1359, E13618, E13620, E13621, E13622, E13628, E13630, E13638, E13649, E1365, E1369
Uncontrolled Diabetes[8]	250.02, 250.03	E10649, E1065, E1165, E11649, E13649, E1365
Lower Extremity Ulcers, Inflammation, and Infection[9]	454.0, 707.1, 680.6, 680.7, 681.1, 682.6, 682.7, 711.05, 711.06, 711.07, 730.05,730.06, 730.07, 730.15, 730.16, 730.17, 730.25, 730.26, 730.27, 730.35, 730.36, 730.37, 730.85, 730.86, 730.87, 730.95, 730.96, 730.97, 785.4	L97, I83.009, I83.019, 183.029, L02.429, L02.439, L02.629, L02.639, L03.039, L03.049, L03.129, L03.119, M00.059, M00.159, M00.259, M00.859, M00.069, M00.169, M00.269, M00.869, M00.079, M00.179, M00.279, M00.879, M86.159, M86.259, M86.169, M86.269, M86.179, M86.279, M86.659, M86.669, M86.679, M86.9, M90.859, M90.869, M90.879, I96

Diabetes-specific discharge records were defined by any record with any of the above listed codes on primary diagnosis.

© 2022 Uppal TS et al. *JAMA Network Open.*

eAppendix 2. eMethods Calculating Crude Rates, Age-Standardized Rates, and Standardized Rate Ratios

Estimating Analogous Denominator Population Estimates using the American Community Survey

Identifying analogous population figures to match subgroup populations broken down by rural/urban status, race, and region in the HCUP data was straightforward. To align insurance definitions with ACS population denominator estimates, if an ACS participant had multiple insurance providers, we considered their age, disability status, and employment status to determine likely primary payer based on a 2018 Medicare User Guide.[10] We recoded insurance categorization in HCUP to align with the ACS estimates using coding guidelines provided by methodology reports published by AHRQ.[11]

The IPUMS ACS provided rural/urban variable had high missingness (>10%). We replaced/imputed the variable with the rural-urban continuum codes published by the United States Department of Agriculture Economic Research Center, which allocates all Public Use Microdata Area data to rural/urban categorization based on metro and non-metro population shares.[12]

Calculation of Crude Population Utilization Rates by Subpopulation

NEDS

Counts and standard errors of inpatient and emergency department events generated using NEDS data were weighted and adjusted for complex survey design factors. HCUP provides discharge weights for the NEDS that are used to generate nationally representative estimates of ED utilization. Thus, the crude diabetes-related ED utilization rate for a given demographic/insurance group using NEDS data is given by equation (1),

$$CR_v = 10{,}000 \cdot \frac{\sum_{i=1}^{n} w_i v_i}{N} = 10{,}000 \cdot \frac{S}{N}$$

Where w_i is the discharge weight associated with the patient i, and v_i is the variable of interest (i.e. rural, 1=rural, 0=not rural). N represents the population estimate for the variable of interest.[13] Variance for survey-weighted estimates were estimated using Taylor Series Linearization methodology provided in the *Survey* package for R.[14]

We assumed constant denominator population counts,[13] consequently the standard error of the crude rate was calculated as:

$$SE_R = 10{,}000\, SE_S/N$$

Where SE_s is the standard error of the estimated count.

SEDD

Although the SEDD are considered a census of visits to hospitals in a given state, because we are estimating age-standardized estimates and are thus describing a different estimate than the direct rate of use within a state for a given year, we considered the state databases to be samples from a "super-population" for the purposes of variance estimation. We assumed a Poisson distribution of counts for purpose of variance estimation.[15,16] The state databases are not weighted as each file contains nearly all discharges from nearly all hospitals within a given state, thus the crude ED utilization rate using SEDD data is given by equation (2),

$$R = 10{,}000 \cdot \frac{S}{N}$$

Where S is the number of estimated counts, and N is the corresponding denominator population eFigure. We assumed constant denominator population counts, consequently the standard error of rates was calculated as:

$$SE_R = 10{,}000\, SE_S/N$$

Where SE_s is the standard error of the estimated count.

© 2022 Uppal TS et al. *JAMA Network Open.*

Standardization of Rates

Standardized rates were calculated using the direct method. Standardized rates reflect rates that would be expected for the observed study population if it had the same population distribution as the standard population.

Standard Population

All rates excluding age-specific and insurance-specific rates are age standardized using standard population distribution of the U.S. Adult Population. Standard population data of national age distribution was collected from the 2010 CDC National Mortality Database. We estimated the observed crude rate for each of the for each of the following age-specific bins for each demographic group: 18-29, 30-44, 45-64, 65-74, 75+. Standard population estimates for the age distribution in the United States were generated from the 2010 CDC National Mortality Database.[17]

Calculating Standardized Rates and Standardized Rate Error Estimates

We calculated the weighted average of bin-specific observed rates using weights proportional to the percentage of the standard population in age-specific cell.

$$R_{std} = Standardized\ Rate = \sum_i w_i P_i$$

Where *i* = 1 to 5 standard population bins, w_i is the proportion of the standard population in population group *i* of the total standard population and P_i is the observed age-specific crude rate.[18]

The variance of standardized rates was calculated as:

$$var_{std} = Variance\ (Standardized\ Rate) = \sum_i {w_i}^2 * Variance\ (Pi)$$

Where *i* = 1 to 5 standard population bins, w_i is the proportion of the standard population in population group *i* of the total standard population, and P_i is the estimated age-specific crude rate.

95% confidence intervals for standardized rates were calculated as:

$$(R_{std} - 1.96 * \sqrt{var_{std}}\ \ ,\ R_{std} + 1.96 * \sqrt{var_{std}})$$

Age and Insurance Specific Rates

Age-specific rates and insurance-specific rates were not standardized because of the age-distributions within these groups. Insurance-specific crude rates were reported because direct standardization is unstable in the case of a zero-cell count (i.e., # of diabetes-specific ED visits in Vermont, ages 18-29, Medicare population). Therefore, insurance-specific rates do not control for population age composition and variability between states in these rates may be a result of variation in age distribution between geographies.[19]

Rate Ratios

To compare rates of ED, use between groups and by diabetes status, we calculated rate ratios.

$$RR = \frac{R_1}{R_2}$$

Where R_1 is the rate being compared and R_2 is the reference rate. Confidence intervals for standardized rate ratios were then estimated assuming a log-normal distribution.[20] We generated 95% confidence intervals around our rate ratios by first estimating the variance of the RR:

$$Var[\log(RR)] = \frac{Var(R_1)}{(R_1)^2} + \frac{Var(R_2)}{(R_2)^2}$$

95% confidence intervals for SRRs were calculated as:

$$\left(\exp\left(\log(RR) - 1.96 * \sqrt{Var[\log(RR)]}\right),\ \exp\left(\log(RR) + 1.96 * \sqrt{Var[\log(RR)]}\right)\right)$$

© 2022 Uppal TS et al. *JAMA Network Open.*

eTable 5. Sample Characteristics of All-Cause and Diabetes-Specific ED Visits From the National Emergency Department Dataset, Combined Data From Years 2008, 2011, 2014, 2016[a]

Variable[b]	All-Cause Diabetes ED Visits		Diabetes-Specific ED Visits	
# of Visits				
Weighted	32,433,015		1,911,795	
(se)	(658,143)		(38,228)	
Age (Mean)	58.4		55.0	
(sd)	(16.3)		(17.4)	
Age Groups				
18-29	4.6	(4.5, 4.7)	8.7	(8.5, 8.9)
30-44	15.9	(15.7, 16.1)	19.4	(19.2, 19.7)
45-64	42.6	(42.3, 42.8)	41.2	(40.9, 41.5)
65-74	17.1	(17, 17.3)	14.2	(14, 14.4)
75+	19.9	(19.5, 20.2)	16.5	(16.1, 16.8)
% Female	56.8	(56.5, 57)	49.5	(49.2, 49.8)
% Rural	20.0	(18.9, 21)	21.6	(20.6, 22.7)
Region				
Midwest	22.9	(21.3, 24.5)	22.1	(20.6, 23.6)
Northeast	14.5	(13, 15.9)	15.7	(14.2, 17.1)
South	43.5	(41.5, 45.5)	41.9	(39.9, 43.9)
West	19.2	(17.8, 20.6)	20.4	(19, 21.8)
Insurance				
Medicaid	18.3	(17.8, 18.8)	20.8	(20.2, 21.4)
Medicare	46.6	(46.1, 47.1)	41.9	(41.3, 42.4)
Private	3.4	(3.2, 3.7)	21.9	(21.4, 22.4)
Uninsured	22.6	(22.1, 23.1)	12.3	(11.7, 12.8)
Other	9.1	(8.6, 9.5)	3.2	(2.9, 3.5)

[a] Dataset generated from National Emergency Department Sample data, years 2008, 2011, 2014, and 2016. All-cause diabetes visits include all discharge records with a diabetes-related diagnosis while diabetes-specific visits include all visits with a principal diagnosis of a diabetes specific condition/complication.

[b] Continuous variables reported as mean (sd) and proportions presented as proportion (95% C.I.)

© 2022 Uppal TS et al. *JAMA Network Open.*

eAppendix 3. Analysis by Diabetes Status

We also estimated rates of ED use among people with diabetes and without diabetes using the NEDS. Diabetes status was determined in NEDS if any recorded discharge diagnosis code had an ICD-9 code (250.XX) or ICD-10 (E10.XXX, E11.XXX, E13.XXX) code indicative of diabetes. Events consistent with both diabetes diagnosis and ages >=18 were selected. We estimated health-care use among those without diabetes by selecting all discharge records which did not have presence of an ICD-9 or ICD-10 code indicative of diabetes. The denominator population estimates were generated using CDC's Behavioral Risk Factor Surveillance Survey (BRFSS), which includes a variable defining self-reported diabetes status. We used the BRFSS to estimate the national US adult population with and without diabetes.

All rates are age- direct standardized using a standard population distribution of the U.S. Adult Population in 2010 using the 2010 US Adult Population using estimates available from the CDC Mortality Database. Standardized rate ratios (SRRs) were calculated across years, using 2008 service utilization rates as the reference. SRRs were also calculated by diabetes status, using non-diabetes service utilization rates as the reference.

eTable 6. Age Adjusted Rates of Average Annual Recorded Emergency Department Visits (per 1000 Adults) Among US Adult Population With and Without Diabetes From 2008-2016

	Rates (95% C.I)							
	2008		2011		2014		2016	
Diabetes								
Rate of ED visits	343.6	(333.4, 353.9)	398.7	(387.5, 410.0)	440.5	(425.8, 455.1)	498.7	(482.3, 515.2)
Ratio[b]	-		1.16	(1.15, 1.17)	1.28	(1.27, 1.30)	1.45	(1.44, 1.47)
Non-Diabetes								
Rate of ED visits	343.6	(335.8, 351.5)	363.1	(355.4, 370.9)	371.5	(361.9, 381.1)	381.5	(370.9, 392.1)
Ratio[b]	-		1.05	(1.04, 1.07)	1.08	(1.07, 1.09)	1.11	(1.10, 1.12)
	Rate Ratios (95% C.I)							
Diabetes/Non-Diabetes[c]								
Rate of ED visits	1.00	(0.99, 1.01)	1.1	(1.09, 1.11)	1.19	(1.17, 1.19)	1.31	(1.29, 1.32)

[a]All rates are directly-age standardized to the 2010 US Adult Population without Diabetes, using weighted estimates generated from the 2010 Behavioral Risk Factor Surveillance Survey
[b] Ratios reflect rate ratios and 95% C.I. using 2008 rates as reference
[c] Reflects rate ratios using non-Diabetes healthcare utilization rates as reference

eTable 6 shows the rates of all-cause national ED visits per 1000 persons by diabetes status for years 2008, 2011, 2014, and 2016. From 2008 to 2016, the rates of ED service use have increased in the non-diabetes population (2016 RR: 1.11; 95% CI, 1.10, 1.12) and the diabetes population (2016 RR: 1.45; 95% CI, 1.44, 1.47), with greater increases in the diabetes population. The rate ratio of ED service use among people with diabetes compared to the non-diabetes population has increased from 2008, where rates of ED use resulting in discharge were nearly identical in both populations (RR: 1.00, 95% CI, 0.99, 1.01) to 2016 (2016 RR: 1.31; 95% CI, 1.29, 1.32).

© 2022 Uppal TS et al. *JAMA Network Open.*

eTable 7. Rate Ratio of Average Annual Diabetes-Specific Emergency Department Visits (per 10,000 Adults) Among US Adult Population From 2008-2017[a]

	2008		2011		2014		2016 / 2017[b]		Average, 2008-2017	
	Rate Ratio	(95% CI)	Rate Ratio	(95% CI)	Rate Ratio	(95% CI)	Rate Ratio	(95% CI)	Rate Ratio	(95% CI)
Race (Ref. White, non-Hispanic)[c]										
Black, non-Hispanic	3.03	(2.71, 3.39)	3.06	(2.78, 3.38)	2.88	(2.63, 3.16)	3.18	(3.00, 3.38)	3.09	(2.91, 3.30)
Hispanic	1.34	(1.16, 1.53)	1.10	(0.97, 1.24)	1.22	(1.09, 1.36)	1.28	(1.19, 1.38)	1.29	(1.19, 1.40)
Rural/Urban (Ref. Urban)[d]										
Rural	1.43	(1.30, 1.58)	1.42	(1.29, 1.56)	1.28	(1.16, 1.40)	1.18	(1.11, 1.26)	1.34	(1.26, 1.44)
Insurance (Ref. Private)										
Medicaid	5.05	(4.86, 5.26)	6.97	(6.73, 7.21)	6.57	(6.35, 6.78)	6.75	(6.09, 7.49)	6.65	(6.49, 6.82)
Medicare	4.24	(4.06, 4.42)	5.32	(5.10, 5.54)	4.67	(4.51, 4.84)	3.78	(3.40, 4.20)	4.37	(4.23, 4.51)
Uninsured	1.94	(1.79, 2.10)	2.35	(2.17, 2.54)	2.19	(2.04, 2.36)	2.87	(2.57, 3.20)	2.25	(2.12, 2.40)

[a]Average ED visits aggregated from pooled SEDD data from states AZ, FL, IA, KY, MD, NC, NE, NJ, NY, UT, VT. Population estimates generated from pooled state populations estimated from the IPUMS USA American Community Survey for corresponding years. All rates except insurance were age-standardized to the 2010 US Adult

[b]Rate ratio calculated from an average ED utilization rates from 2016 and 2017 estimates, as 2017 data was not available for select states.

[c]Average use across states for Race estimates do not include NE and VT due to missing race variable, or low # of events

[d]Average use across states for Rural/Urban estimates do not include NJ due to lack of rural counties as defined by USDA supplied rural/urban variable

© 2022 Uppal TS et al. *JAMA Network Open.*

eTable 8. Rates of Diabetes-Specific Emergency Department Visits per 10,000 Adults in Arizona; Years 2008-2017

Variable	2008				2011				2014				2017				2017-2008		
	# of E.D. Visits	Denominator	Rate	(c.i.)	# of E.D. Visits	Denominator	Rate	(c.i.)	# of E.D. Visits	Denominator	Rate	(c.i.)	# of E.D. Visits	Denominator	Rate	(c.i.)	Absolute Δ	Percent Δ	(c.i.)
Age																			
18-29	457	1,077,305	4.2	(3.9,4.6)	593	1,087,985	5.5	(5,5.9)	631	1,116,039	5.7	(5.2,6.1)	1,522	1,171,053	13.0	(12.3,13.6)	8.8	209.5	(195.3, 223.7)
30-44	1,329	1,319,581	10.1	(9.5,10.6)	1,366	1,249,964	10.9	(10.3,11.5)	1,659	1,294,687	12.8	(12.2,13.4)	3,234	1,318,320	24.5	(23.7,25.4)	14.4	142.6	(134.4, 150.8)
45-64	2,201	1,519,215	14.5	(13.9,15.1)	2,759	1,599,974	17.2	(16.6,17.9)	3,369	1,628,864	20.7	(20,21.4)	6,001	1,697,501	35.4	(34.5,36.2)	20.9	144.1	(137.5, 150.7)
65-74	778	412,979	18.8	(17.5,20.2)	952	482,729	19.7	(18.5,21)	1184	577,502	20.5	(19.3,21.7)	1,946	645,040	30.2	(28.8,31.5)	11.4	60.6	(51.9, 69.3)
75+	1,024	447,485	22.9	(21.5,24.3)	1,050	439,111	23.9	(22.5,25.4)	1,217	493,146	24.7	(23.3,26.1)	1,765	552,520	31.9	(30.5,33.4)	9.0	39.3	(31.6, 47)
Sex																			
Female	2,751	2,405,053	11.2	(10.8,11.6)	3,185	2,463,272	12.6	(12.2,13)	3,741	2,597,140	14.0	(13.5,14.4)	6,964	2,730,465	25.2	(24.6,25.8)	14.0	125.0	(119.7, 130.3)
Male	3,038	2,371,512	13.2	(12.7,13.7)	3,535	2,396,491	15.0	(14.5,15.5)	4,319	2,513,098	17.3	(16.8,17.9)	7,504	2,653,969	28.6	(27.9,29.2)	15.4	116.7	(112.2, 121.2)
Race/Ethnicity																			
White, Non-Hispanic	-	-	-	-	-	-	-	-	4469	3123174	13.3	(12.9,13.8)	7,096	3,201,040	21.3	(20.8,21.8)	-	-	-
Black, Non-Hispanic	-	-	-	-	-	-	-	-	546	202353	30.1	(27.5,32.6)	1,379	219,796	67.8	(64.1,71.4)	-	-	-
Hispanic, any race	-	-	-	-	-	-	-	-	2264	1353068	21.0	(20.1,21.9)	4,730	1,480,422	37.5	(36.4,38.6)	-	-	-
Rural/Urban																			
Rural	941	460,355	19.4	(18.2,20.7)	1,112	470,682	22.1	(20.8,23.4)	681	529,619	12.0	(11,12.9)	926	535,281	16.6	(15.5,17.8)	-2.8	-14.4	(-21.6, -7.2)
Urban	4,848	4,316,210	11.3	(11,11.6)	5,608	4,389,081	12.8	(12.5,13.1)	7,379	4,580,619	16.1	(15.7,16.4)	13,542	4,849,153	28.0	(27.6,28.5)	16.7	147.8	(143.7, 151.9)
Insurance																			
Medicaid	1,497	337,252	44.4	(42.1,46.6)	1,871	471,844	39.7	(37.9,41.4)	2,528	496,345	50.9	(48.9,52.9)	5,830	594,676	98.0	(95.5,100.6)	53.6	120.7	(114.4, 127)
Medicare	2,295	945,447	24.3	(23.3,25.3)	2,745	1,018,680	26.9	(25.9,28)	3,271	1,171,311	27.9	(27,28.9)	4,995	1,294,597	38.6	(37.5,39.7)	14.3	58.8	(54, 63.6)
Private	1,315	2,445,558	5.4	(5.1,5.7)	1,201	2,295,628	5.2	(4.9,5.5)	1,473	2,500,398	5.9	(5.6,6.2)	2,464	2,714,220	9.1	(8.7,9.4)	3.7	68.5	(61.7, 75.3)
Uninsured	506	874,954	5.8	(5.3,6.3)	733	894,434	8.2	(7.6,8.8)	650	744,112	8.7	(8.1,9.4)	829	583,927	14.2	(13.2,15.2)	8.4	144.8	(128.2, 161.4)
Total	5,789	4,776,565	12.1	(11.8,12.5)	6,720	4,859,763	13.8	(13.4,14.1)	8,060	5,110,238	15.6	(15.3,16)	14,468	5,384,434	26.9	(26.4,27.3)	14.8	122.3	(118.4, 126.2)

[a] Total # of ED Visits N=35,037

[b] All rates except Age-Specific and Insurance-Specific rates are age-standardized to the 2010 US Adult Population using data from the CDC Mortality Database

[c] The transition from ICD-9 CM/PCS to ICD-10 CM/PCS coding occured in third quarter of 2015. Estimates reported from years post-2015 were estimated using ICD-10 codes and may not be directly comparable to rates

[d] Value of - indicates estimate was not reported. All estimates n<=10 events or RSE>=30% were not reported. Additionally, HCUP prioritizes ethnicity when coding the uniform Race variable, AZ did not provide an ethnicity variable in 2008 and 2011.

© 2022 Uppal TS et al. *JAMA Network Open.*

eTable 9. Rates of Diabetes-Specific Emergency Department Visits per 10,000 Adults in Florida; Years 2008-2017

	2008				2011				2014				2017				2017-2008		
Variable	# of E.D. Visits	Denominator	Rate	(c.i.)	# of E.D. Visits	Denominator	Rate	(c.i.)	# of E.D. Visits	Denominator	Rate	(c.i.)	# of E.D. Visits	Denominator	Rate	(c.i.)	Absolute △	Percent △	(c.i.)
Age																			
18-29	1509	2,783,157	5.4	(5.1,5.7)	1723	2,985,810	5.8	(5. 5,6)	1920	3,080,075	6.2	(6, 6.5)	4,111	3,158,262	13.0	(12.6 ,13.4)	7.6	141.1	(134.3, 147.9)
30-44	3,613	3,590,210	10.1	(9.7,10.4)	4,025	3,545,422	11.4	(11, 11.7)	4,642	3,668,380	12.7	(12.3, 13)	8,874	3,857,687	23.0	(22.5, 23.4)	12.9	127.7	(122.6, 132.8)
45-64	8,152	4,764,788	17.1	(16.7,17.5)	9,743	5,173,375	18.8	(18.5, 19.2)	10,937	5,296,412	20.6	(20.3, 21)	18,759	5,554,964	33.8	(33.3, 34.3)	16.7	97.5	(94.4, 100.6)
65-74	3026	1,419,169	21.3	(20.6,22.1)	3440	1,642,006	20.9	(20.2,21.7)	3712	1,928,373	19.2	(18.6, 19.9)	6,076	2,139,804	28.4	(27.7, 29.1)	7.1	33.3	(29.1, 37.5)
75+	4,496	1,762,049	25.5	(24.8,26.3)	4,463	1,718,192	26.0	(25.2,26.7)	4,352	1,868,050	23.3	(22.6, 24)	6,439	2,074,993	31.0	(30.9 ,31.8)	5.5	21.7	(18.1, 25.3)
Sex																			
Female	10,427	7,374,758	13.1	(12.8,13.3)	11,646	7,787,443	14.0	(13.7,14.2)	12,632	8,192,489	14.6	(14.3, 14.9)	21,767	8,671,836	24.4	(24.0, 24.7)	11.3	86.1	(83.6, 88.6)
Male	10,369	6,944,615	14.5	(14.2,14.8)	11,748	7,277,362	15.7	(15.4,15.9)	12,931	7,648,801	16.4	(16.2, 16.7)	22,492	8,113,874	27.1	(26.8, 27.5)	12.6	87.2	(84.9, 89.5)
Race/Ethnicity																			
White, Non-Hispanic	-	-	-	-	12103	9124354	11.9	(11.7,12.2)	12628	9299079	12.5	(12.3, 12.8)	20,361	9,542,674	20.5	(20.2, 20.8)	-	-	-
Black, Non-Hispanic	-	-	-	-	7173	2116845	37.8	(36.9,38.7)	7643	2272110	36.3	(35.4, 37.1)	14,640	2,399,728	63.9	(62.9, 65.0)	-	-	-
Hispanic	-	-	-	-	3571	3232996	12.0	(11.6,12.4)	4593	3598495	13.5	(13.1, 13.9)	8,136	4,062,171	20.6	(20.2, 21.1)	-	-	-
Rural/Urban																			
Rural	1,847	1,020,740	16.9	(16.1,17.7)	2,050	1,051,316	17.8	(17,18.6)	1,474	963,272	14.5	(13.7, 15.2)	2361	998,650	23.5	(22.5, 24.5)	6.6	39.0	(32.7, 45.3)
Urban	18,949	13,298,633	13.5	(13.3,13.7)	21,344	14,013,489	14.5	(14.3,14.7)	24,089	14,878,018	15.5	(15.3, 15.7)	41,898	15,787,060	25.8	(25.6, 26.1)	12.3	91.3	(89.3, 93.3)
Insurance																			
Medicaid	2,366	519,576	45.5	(43.7,47.4)	3,843	790,678	48.6	(47.1,50.1)	4,333	973,616	44.5	(43.2, 45.8)	8,079	1,010,681	79.9	(78.2, 81.7)	34.4	75.7	(71.1, 80.3)
Medicare	9,504	3,436,830	27.7	(27.1,28.2)	10,582	3,674,467	28.8	(28.3,29.3)	11,321	4,124,206	27.5	(26.9, 28)	17,189	4,525,869	38.0	(37.4, 38.6)	10.3	37.1	(34.6, 39.6)
Private	4,045	6,973,815	5.8	(5.6,6)	3,531	6,712,132	5.3	(5.1,5.4)	4,388	7,392,875	5.9	(5.8, 6.1)	8,866	8,391,459	10.6	(10.4, 10.8)	4.8	82.2	(78, 86.4)
Uninsured	4088	3,117,459	13.1	(12.7,13.5)	4583	3,570,343	12.8	(12.5,13.2)	4558	3,000,154	15.2	(14.8, 15.6)	8633	2,506,717	34.4	(33.7 , 35.2)	21.3	162.9	(157.6, 168.2)
Total	20,796	14,319,373	13.8	(13.6,13.9)	23,394	15,064,805	14.8	(14.6,15)	25,563	15,841,290	15.5	(15.3, 15.7)	44,259	16,785,710	25.7	(25.4, 25.9)	11.9	86.0	(84.1, 87.9)

[a] Total # of ED Visits N=114,012

[b] All rates except Age-Specific and Insurance-Specific rates are age-standardized to the 2010 US Adult Population using data from the CDC Mortality Database

[c] The transition from ICD-9 CM/PCS to ICD-10 CM/PCS coding occured in third quarter of 2015. Estimates reported from years post-2015 were estimated using ICD-10 codes and may not be directly comparable to rates estimated pre-2015.

[d] Value of - indicates estimate was not reported. All estimates n<=10 events or RSE>=30% were not reported. Additionally, HCUP prioritizes ethnicity when coding the uniform Race variable, FL, IA, and NC did not provide an ethnicity variable in 2008.

© 2022 Uppal TS et al. *JAMA Network Open.*

eTable 10. Rates of Diabetes-Specific Emergency Department Visits per 10,000 Adults in Iowa; Years 2008-2017

	2008				2011				2014				2017				2017-2008		
Variable	# of E.D. Visits	Denominator	Rate	(c.i.)	# of E.D. Visits	Denominator	Rate	(c.i.)	# of E.D. Visits	Denominator	Rate	(c.i.)	# of E.D. Visits	Denominator	Rate	(c.i.)	Absolute △	Percent △	(c.i.)
Age																			
18-29	360	514,831	7.0	(6.3,7.7)	374	508,767	7.3	(6.6,8.1)	376	511,098	7.4	(6.6,8.1)	810	519,925	15.6	(14.5,16.7)	8.6	123.2	(108.5, 137.9)
30-44	676	554,642	12.2	(11.3,13.1)	712	552,221	12.9	(12.0,13.8)	755	561,554	13.4	(12.5,14.4)	1,427	569,426	25.1	(23.8,26.4)	12.9	105.9	(94.5, 117.3)
45-64	1,431	787,644	18.2	(17.2,19.1)	1,565	820,979	19.1	(18.1,20.0)	1,512	817,938	18.5	(17.6,19.4)	2,682	802,022	33.4	(32.2,34.7)	15.2	83.8	(76.8, 90.8)
65-74	568	192,149	29.6	(27.1,32.0)	622	210,963	29.5	(27.2,31.8)	574	241,275	23.8	(21.8,25.7)	903	267,215	33.8	(31.6,36)	4.2	14.3	(5.0, 23.6)
75+	1,064	248,618	42.8	(40.2,45.4)	903	246,990	36.6	(34.2,38.9)	831	250,593	33.2	(30.9,35.4)	977	256,554	38.1	(35.7,40.5)	-4.7	-11.0	(-17.8, -4.2)
Sex																			
Female	2,026	1,178,886	15.9	(15.2,16.6)	2,063	1,193,978	16.2	(15.5,17.0)	1869	1,212,257	14.7	(14.0,15.3)	3,286	1,227,627	26.2	(25.2,27.1)	10.3	64.5	(58.1, 70.9)
Male	2,073	1,118,998	18.7	(17.9,19.5)	2,113	1,145,942	18.4	(17.6,19.2)	2179	1,170,201	18.4	(17.6,19.1)	3,513	1,187,515	29.5	(28.5,30.4)	10.8	57.9	(52.2, 63.6)
Race/Ethnicity																			
Hispanic	-	-	-	-	3713	2118989	16.5	(16.0,17)	3523	2131366	15.5	(15.0,16.1)	5,604	2,128,722	25.4	(24.8,26.1)	-	-	-
Hispanic	-	-	-	-	297	53633	69.2	(60.7,77.7)	298	68893	51.4	(45.3,57.5)	688	67,976	111.5	(102.9,120.1)	-	-	-
Hispanic	-	-	-	-	91	90769	14.6	(10.9,18.4)	146	102599	21.0	(17.0,25.1)	268	114,337	35.4	(30.1,40.7)	-	-	-
Rural/Urban																			
Rural	2,106	1,159,789	16.5	(15.7,17.2)	2,052	1,155,609	16.2	(15.5,16.9)	2055	917,459	20.7	(19.8,21.6)	3167	907,571	33.5	(32.3,34.7)	17.0	103.5	(96.4, 110.6)
Urban	1,993	1,138,095	17.9	(17.1,18.7)	2,124	1,184,311	18.4	(17.6,19.2)	1993	1,464,999	13.6	(13.0,14.2)	3,632	1,507,571	24.3	(23.5,25.1)	6.4	35.5	(30.3, 40.7)
Insurance																			
Medicaid	466	96,410	48.3	(44.0,52.7)	654	114,769	57.0	(52.6,61.4)	887	161,225	55.0	(51.4,58.6)	2,048	197,985	103.4	(99, 107.9)	55.1	113.9	(102.9, 124.9)
Medicare	2,132	487,455	43.7	(41.9,45.6)	2,077	509,930	40.7	(39.0,42.5)	2025	551,939	36.7	(35.1,38.3)	3,032	573,828	52.8	(51,54.7)	9.1	20.7	(15.5, 25.9)
Private	1,032	1,472,585	7.0	(6.6,7.4)	977	1,443,397	6.8	(6.45,7.2)	900	1,489,539	6.0	(5.7,6.4)	1,428	1,498,814	9.5	(9.0,10.0)	2.5	35.5	(26.7, 44.3)
Uninsured	382	221,821	17.2	(15.5,19.0)	370.4	244,747	15.1	(13.6,16.7)	201	159,690	12.6	(10.9,14.3)	231	118,942	19.4	(16.9,21.9)	2.2	12.7	(-2.3, 27.7)
Total	4,099	2,297,884	17.2	(16.6,17.7)	4,176	2,339,920	17.2	(16.7,17.8)	4,048	2,382,458	16.4	(15.9,16.9)	6,799	2,415,142	27.7	(27.1,28.4)	10.6	61.5	(57.6, 65.4)

[a] Total # of ED Visits N=19,122

[b] All rates except Age-Specific and Insurance-Specific rates are age-standardized to the 2010 US Adult Population using data from the CDC Mortality Database

[c] The transition from ICD-9 CM/PCS to ICD-10 CM/PCS coding occured in third quarter of 2015. Estimates reported from years post-2015 were estimated using ICD-10 codes and may not be directly comparable to rates estimated pre-2015.

[d] Value of - indicates estimate was not reported. All estimates n<=10 events or RSE>=30% were not reported. Additionally, HCUP prioritizes ethnicity when coding the uniform Race variable, FL, IA, and NC did not provide an ethnicity variable in 2008.

© 2022 Uppal TS et al. *JAMA Network Open.*

eTable 11. Rates of Diabetes-Specific Emergency Department Visits per 10,000 Adults in Kentucky; Years 2008-2017

	2008			2011			2014			2017			2017-2008		
Variable	# of E.D. Visits	Denominator	Rate (c.i.)	# of E.D. Visits	Denominator	Rate (c.i.)	# of E.D. Visits	Denominator	Rate (c.i.)	# of E.D. Visits	Denominator	Rate (c.i.)	Absolute △	Percent △	(c.i.)
Age															
18-29	570	686,355	8.3 (7.6,9)	732	701,845	10.4 (9.7,11.2)	697	705,491	9.9 (9.1,10.6)	1,472	723,004	20.4 (19.3,21.4)	12.1	145.8	(134.2, 157.4)
30-44	1,335	875,762	15.2 (14.4,16.1)	1,638	854,038	19.2 (18.3,20.1)	1,910	844,789	22.6 (21.6,23.6)	3,078	828,086	37.2 (35.9,38.5)	22.0	144.7	(135.9, 153.5)
45-64	2,510	1,141,903	22.0 (21.1,22.8)	3,144	1,197,727	26.2 (25.3,27.2)	3,535	1,195,181	29.6 (28.6,30.6)	5,982	1,181,324	50.6 (49.4,51.9)	28.6	130.0	(124, 136)
65-74	969	282,371	34.3 (32.2,36.5)	1041	310,591	33.5 (31.5,35.6)	1188	361,611	32.9 (31,34.7)	1,705	388,350	43.9 (41.8,46)	9.6	28.0	(20.5, 35.5)
75+	1,143	278,395	41.1 (38.7,43.4)	1,159	283,281	40.9 (38.6,43.3)	1,158	292,355	39.6 (37.3,41.9)	1,564	320,578	48.8 (46.4,51.2)	7.7	18.7	(11.9, 25.5)
Sex															
Female	3,394	1,687,215	19.4 (18.8,20.1)	3,970	1,723,771	22.4 (21.7,23.1)	4,318	1,745,872	23.9 (23.2,24.7)	6,848	1,769,085	38.1 (37.2,39)	18.7	96.4	(91.4, 101.4)
Male	3,133	1,577,571	20.2 (19.5,21)	3,744	1,623,711	23.3 (22.5,24)	4,170	1,653,555	25.1 (24.4,25.9)	6,953	1,672,257	41.5 (40.5,42.5)	21.3	105.4	(100.2, 110.6)
Race/Ethnicity															
White, Non-Hispanic	5421	2,914,443	18.2 (17.7,18.7)	6532	2,936,726	21.7 (21.2,22.2)	7132	2,963,813	23.4 (22.8,23.9)	11,427	2,974,103	37.7 (37,38.4)	19.5	107.1	(103, 111.2)
Black, Non-Hispanic	831	228,993	40.5 (37.7,43.4)	1093	256,770	47.2 (44.4,50.1)	1075	257,591	44.3 (41.6,47)	2,078	267,804	80.2 (76.7,83.6)	39.7	98.0	(88.7, 107.3)
Hispanic	31	60,828	9.3 (5.1,13.5)	48	81,175	8.4 (5.7,11)	148	89,378	29.0 (23.4,34.7)	188	95,903	29.0 (23.9,34.1)	19.7	211.8	(151.4, 272.2)
Rural/Urban															
Rural	3,128	1,456,541	21.0 (20.3,21.8)	3,728	1,450,065	25.0 (24.2,25.8)	4,328	1,301,804	32.2 (31.2,33.1)	6829	1,300,430	51.3 (50.1,52.5)	30.3	144.3	(138.6, 150)
Urban	3,399	1,808,245	18.9 (18.2,19.5)	3,986	1,897,417	21.0 (20.4,21.7)	4,160	2,097,623	19.7 (19.1,20.3)	6,972	2,140,912	32.5 (31.7,33.3)	13.6	72.0	(67.6, 76.4)
Insurance															
Medicaid	1,097	200,703	54.7 (51.4,57.9)	1,448	212,517	68.1 (64.6,71.6)	2,499	375,297	66.6 (64,69.2)	5,089	512,109	99.4 (96.6,102.1)	44.7	81.7	(75.1, 88.3)
Medicare	2,904	701,873	41.4 (39.9,42.9)	3,475	761,230	45.6 (44.1,47.2)	3,738	819,549	45.6 (44.1,47.1)	5,478	871,862	62.8 (61.2,64.5)	21.4	51.7	(47.2, 56.2)
Private	1,589	1,788,932	8.9 (8.4,9.3)	1,402	1,709,024	8.2 (7.8,8.6)	1,736	1,776,836	9.8 (9.3,10.2)	2,508	1,773,309	14.1 (13.6,14.7)	5.2	58.4	(51.7, 65.1)
Uninsured	769	513,674	15.0 (13.9,16)	1316	592,445	22.2 (21,23.4)	371	361,709	10.3 (9.2,11.3)	429	217,001	19.8 (17.9,21.6)	4.8	32.0	(19.7, 44.3)
Total	6,527	3,264,786	19.9 (19.4,20.3)	7,714	3,347,482	22.8 (22.2,23.3)	8,488	3,399,427	24.5 (24,25)	13,801	3,441,342	39.7 (39,40.3)	19.8	99.5	(96.5, 102.5)

[a] Total # of ED Visits N=36,530

[b] All rates except Age-Specific and Insurance-Specific rates are age-standardized to the 2010 US Adult Population using data from the CDC Mortality Database

[c] The transition from ICD-9 CM/PCS to ICD-10 CM/PCS coding occured in third quarter of 2015. Estimates reported from years post-2015 were estimated using ICD-10 codes and may not be directly comparable to rates estimated pre-2015.

© 2022 Uppal TS et al. *JAMA Network Open.*

eTable 12. Rates of Diabetes-Specific Emergency Department Visits per 10,000 Adults in Maryland; Years 2008-2017

	2008			2011			2014			2017			2017-2008		
Variable	# of E.D. Visits	Denominator	Rate (c.i.)	# of E.D. Visits	Denominator	Rate (c.i.)	# of E.D. Visits	Denominator	Rate (c.i.)	# of E.D. Visits	Denominator	Rate (c.i.)	Absolute △	Percent △	(c.i.)
Age															
18-29	468	925,017	5.1 (4.6,5.5)	594	961,324	6.2 (5.7,6.7)	643	981,853	6.6 (6.0,7.1)	1,205	956,139	12.6 (11.9,13.3)	7.5	147.1	(132.6, 161.6)
30-44	1,252	1,179,901	10.6 (10,11.2)	1,290	1,168,164	11.0 (10.4,11.6)	1,553	1,184,647	13.1 (12.5,13.8)	2,536	1,197,106	21.2 (20.4,22)	10.6	100.0	(92.2, 107.8)
45-64	2,644	1,509,065	17.5 (16.9,18.2)	3,048	1,622,154	18.8 (18.1,19.5)	3,870	1,643,919	23.5 (22.8,24.3)	5,880	1,653,684	35.6 (34.6,36.5)	18.1	103.4	(97.9, 108.9)
65-74	898	336,994	26.6 (24.9,28.4)	967	376,339	25.7 (24.1,27.3)	1,240	445,619	27.8 (26.3,29.4)	1,794	488,375	36.7 (35,38.4)	10.1	38.0	(30.1, 45.9)
75+	1,213	347,770	34.9 (32.9,36.8)	1,176	354,276	33.2 (31.3,35.1)	1,310	373,097	35.1 (33.2,37.0)	1,767	411,753	42.9 (40.9,44.9)	8.0	22.9	(16.2, 29.6)
Sex															
Female	3,284	2,254,179	14.3 (13.8,14.8)	3,566	2,347,748	14.8 (14.3,15.3)	4,205	2,421,400	16.8 (16.3,17.3)	6,446	2,457,969	25.3 (24.7,25.9)	11.0	76.9	(72, 81.8)
Male	3,191	2,044,568	16.2 (15.7,16.8)	3,509	2,134,509	16.9 (16.3,17.4)	4,411	2,207,735	20.2 (19.6,20.8)	6,736	2,249,088	29.8 (29.1,30.6)	13.6	84.0	(78.9, 89.1)
Race/Ethnicity															
Hispanic	2,851	2,555,841	10.5 (10.1,10.9)	3,347	2,542,349	12.3 (11.9,12.7)	3,662	2,540,532	13.3 (12.9,13.8)	4,894	2,504,317	18.3 (17.8,18.8)	7.8	74.3	(68.6, 80)
Hispanic	3,365	1,199,144	30.5 (29.5,31.6)	3,390	1,269,315	28.4 (27.4,29.4)	4,368	1,325,983	34.0 (33.0,35.1)	7,169	1,367,328	52.9 (51.6,54.1)	22.4	73.4	(69.2, 77.6)
Hispanic	110	248,620	5.6 (4.4,6.8)	132	333,408	6.1 (4.9,7.2)	281	377,452	11.0 (9.5,12.5)	642	408,745	20.9 (19,22.8)	15.3	273.2	(238.8, 307.6)
Rural/Urban															
Rural	571	256,663	22.1 (20.3,23.9)	697	264,105	25.3 (23.4,27.1)	484	286,834	16.0 (14.6,17.4)	550	291,832	17.8 (16.3,19.4)	-4.3	-19.5	(-28.5, -10.5)
Urban	5,904	4,042,084	14.7 (14.4,15.1)	6,378	4,218,152	15.1 (14.8,15.5)	8,132	4,342,301	18.6 (18.2,19.0)	12,632	4,415,225	28.1 (27.6,28.6)	13.4	91.2	(87.2, 95.2)
Insurance															
Medicaid	946	146,118	64.7 (60.6,68.9)	1,405	259,084	54.2 (51.4,57.1)	2,286	381,128	60.0 (57.5,62.4)	3,972	425,596	93.3 (90.4,96.2)	28.6	44.2	(37.6, 50.8)
Medicare	2,664	736,236	36.2 (34.8,37.6)	2,873	794,757	36.1 (34.8,37.5)	3,495	889,220	39.3 (38,40.6)	4,750	967,979	49.1 (47.7,50.5)	12.9	35.6	(31.1, 40.1)
Private	1,753	2,781,283	6.3 (6,6.6)	1,923	2,790,686	6.9 (6.6,7.2)	2,135	2,819,513	7.6 (7.3,7.9)	3,364	2,879,302	11.7 (11.3,12.1)	5.4	85.7	(78.3, 93.1)
Uninsured	1,031	555,310	18.6 (17.4,19.7)	756	548,093	13.8 (12.8,14.8)	542	443,011	12.2 (11.2,13.3)	815	332,280	24.5 (22.8,26.2)	5.9	31.7	(22.1, 41.3)
Total	6,475	4,298,747	15.2 (14.8,15.6)	7,075	4,482,257	15.8 (15.4,16.1)	8,616	4,629,135	18.4 (18,18.8)	13,182	4,707,057	27.4 (27,27.9)	12.2	80.3	(77.2, 83.4)

[a] Total # of ED Visits N=35,348

[b] All rates except Age-Specific and Insurance-Specific rates are age-standardized to the 2010 US Adult Population using data from the CDC Mortality Database

[c] The transition from ICD-9 CM/PCS to ICD-10 CM/PCS coding occured in third quarter of 2015. Estimates reported from years post-2015 were estimated using ICD-10 codes and may not be directly comparable to rates estimated pre-2015.

© 2022 Uppal TS et al. *JAMA Network Open.*

eTable 13. Rates of Diabetes-Specific Emergency Department Visits per 10,000 Adults in North Carolina; Years 2008-2017

	2008				2011				2014				2017				2017-2008		
Variable	# of E.D. Visits	Denominator	Rate	(c.i.)	# of E.D. Visits	Denominator	Rate	(c.i.)	# of E.D. Visits	Denominator	Rate	(c.i.)	# of E.D. Visits	Denominator	Rate	(c.i.)	Absolute △	Percent △	(c.i.)
Age																			
18-29	1,131	1,506,878	7.5	(7.1,7.9)	1,257	1,579,505	8.0	(7.5,8.4)	1,410	1,629,692	8.7	(8.2,9.1)	2,818	1,675,318	16.8	(16.2,17.4)	9.3	123.7	(115.5, 131.9)
30-44	2,764	1,966,936	14.1	(13.5,14.6)	2,827	1,956,756	14.4	(13.9,15)	3,675	1,964,562	18.7	(18.1,19.3)	5,987	1,964,144	30.5	(29.7,31.3)	16.4	117.1	(111.4, 122.8)
45-64	5,387	2,367,982	22.8	(22.1,23.4)	6,031	2,553,324	23.6	(23,24.2)	7,171	2,599,340	27.6	(26.9,28.2)	12,189	2,704,704	45.1	(44.3,45.9)	22.4	98.2	(94.5, 101.9)
65-74	2,046	576,439	35.5	(34.0,37.0)	2,036	671,783	30.3	(29,31.6)	2,397	811,978	29.5	(28.3,30.7)	3,577	909,997	39.3	(38,40.6)	3.8	10.7	(5.8, 15.6)
75+	2,559	562,439	45.5	(43.7,47.3)	2,331	604,764	38.5	(37,40.1)	2,422	649,047	37.3	(35.8,38.8)	3,315	718,475	46.1	(44.6,47.7)	0.6	1.3	(-3.1, 5.7)
Sex																			
Female	7,561	3,621,428	20.4	(19.9,20.8)	7,746	3,837,059	19.7	(19.2,20.1)	8,849	3,986,470	21.6	(21.1,22)	14,525	4,153,432	34.2	(33.7,34.8)	13.8	67.9	(64.8, 71)
Male	6,326	3,359,246	19.8	(19.3,20.3)	6,736	3,529,073	19.5	(19,20)	8,226	3,668,149	22.6	(22.2,23.1)	13,361	3,819,206	35.1	(34.5,35.7)	15.3	77.5	(74.2, 80.8)
Race/Ethnicity																			
Hispanic	-	-	-	-	7,054	5,012,355	13.4	(13.1,13.7)	8,227	5,142,267	15.2	(14.9,15.6)	12,935	5,276,746	23.6	(23.1,24)	-	-	-
Black, Non-Hispanic	-	-	-	-	6,701	1,525,444	46.8	(45.7,48)	7,565	1,599,624	49.1	(48,50.2)	12,781	1,663,098	78.4	(77,79.7)	-	-	-
Hispanic	-	-	-	-	301	507,001	11.3	(9.6,12.9)	678	543,612	21.8	(19.8,23.8)	1,237	591,627	30.1	(28.1,32.1)	-	-	-
Rural/Urban																			
Rural	5,712	2,169,085	25.5	(24.9,26.2)	5,750	2,265,395	24.3	(23.6,24.9)	5,417	2,284,763	22.8	(22.2,23.4)	8,324	2,319,651	34.9	(34.2,35.7)	9.4	36.7	(33.3, 40.1)
Urban	8,175	4,811,589	17.6	(17.2,18.0)	8,732	5,100,737	17.4	(17.1,17.8)	11,658	5,369,856	21.8	(21.4,22.2)	19,562	5,652,987	34.5	(34.1,35)	16.9	96.1	(93.4, 98.8)
Insurance																			
Medicaid	1,978	313,186	63.2	(60.4,66.0)	2,298	407,701	56.4	(54.1,58.7)	2,853	440,204	64.8	(62.4,67.2)	4,701	487,943	96.3	(93.6,99.1)	33.1	52.5	(47.3, 57.7)
Medicare	6,246	1,352,260	46.2	(45.0,47.3)	6,221	1,522,184	40.9	(39.9,41.9)	6,343	1,709,304	37.1	(36.2,38)	8,577	1,871,776	45.8	(44.9,46.8)	-0.4	-0.8	(-3.5, 1.9)
Private	3,227	3,877,629	8.3	(8.0,8.6)	3,093	3,785,857	8.2	(7.9,8.5)	4,622	4,075,326	11.3	(11,11.7)	8,647	4,344,322	19.9	(19.5,20.3)	11.6	139.2	(134.2, 144.2)
Uninsured	2,151	1,220,469	17.6	(16.9,18.4)	2,548	1,398,146	18.2	(17.5,18.9)	2,910	1,180,182	24.7	(23.8,25.6)	5,309	998,211	53.2	(51.8,54.6)	35.6	201.9	(194.4, 209.4)
Total	13,887	6,980,674	20.1	(19.8,20.4)	14,482	7,366,132	19.6	(19.3,19.9)	17,075	7,654,619	22.0	(21.7,22.4)	27,886	7,972,638	34.6	(34.2,35)	14.5	72.1	(69.9, 74.3)

[a] Total # of ED Visits N=73,330

[b] All rates except Age-Specific and Insurance-Specific rates are age-standardized to the 2010 US Adult Population using data from the CDC Mortality Database

[c] The transition from ICD-9 CM/PCS to ICD-10 CM/PCS coding occured in third quarter of 2015. Estimates reported from years post-2015 were estimated using ICD-10 codes and may not be directly comparable to rates estimated pre-2015.

[d] Value of - indicates estimate was not reported. All estimates n<=10 events or RSE>=30% were not reported. Additionally, HCUP prioritizes ethnicity when coding the uniform Race variable, FL, IA, and NC did not provide an ethnicity variable in

© 2022 Uppal TS et al. *JAMA Network Open.*

eTable 14. Rates of Diabetes-Specific Emergency Department Visits per 10,000 Adults in Nebraska; Years 2008-2017

	2008			2011			2014			2017			2017-2008		
Variable	# of E.D. Visits	Denominator	Rate (c.i.)	# of E.D. Visits	Denominator	Rate (c.i.)	# of E.D. Visits	Denominator	Rate (c.i.)	# of E.D. Visits	Denominator	Rate (c.i.)	Absolute △	Percent △	(c.i.)
Age															
18-29	116	306,221	3.8 (3.1,4.5)	134	315,155	4.3 (3.5,5)	136	310,033	4.4 (3.6,5.1)	327	316,312	10.3 (9.2,11.5)	6.5	171.1	(139.8, 202.4)
30-44	259	335,474	7.7 (6.8,8.7)	243	337,329	7.2 (6.3,8.1)	268	360,994	7.4 (6.5,8.3)	495	364,731	13.6 (12.4,14.8)	5.9	76.6	(59.9, 93.3)
45-64	553	449,350	12.3 (11.3,13.3)	608	480,215	12.7 (11.7,13.7)	689	470,520	14.6 (13.5,15.7)	985	469,395	21 (19.7,22.3)	8.7	70.7	(59.2, 82.2)
65-74	268	107,290	25.0 (22,28)	264	114,778	23.0 (20.2,25.8)	293	137,087	21.4 (18.9,23.8)	408	153,222	26.6 (24,29.2)	1.6	6.4	(-6.7, 19.5)
75+	511	129,899	39.3 (35.9,42.7)	367	135,971	27.0 (24.2,29.8)	325	134,303	24.2 (21.6,26.8)	464	141,352	32.8 (29.8,35.8)	-6.5	-16.5	(-26, -7)
Sex															
Female	851	680,017	11.6 (10.8,12.4)	736	706,151	9.9 (9.1,10.6)	837	717,025	11.3 (10.5,12.1)	1,410	729,285	18.8 (17.8,19.8)	7.2	62.1	(53, 71.2)
Male	856	648,217	13.7 (12.8,14.7)	880	677,297	13.3 (12.4,14.1)	874	695,912	12.7 (11.9,13.5)	1,269	715,727	17.7 (16.7,18.6)	4.0	29.2	(20.7, 37.7)
Rural/Urban															
Rural	948	569,030	14.8 (13.8,15.8)	828	576,261	13.2 (12.3,14.1)	746	576,096	11.8 (10.9,12.7)	1073	574,919	17.3 (16.3,18.4)	2.5	16.9	(9, 24.8)
Urban	759	759,204	10.6 (9.9,11.4)	788	807,187	10.3 (9.6,11)	965	836,841	11.9 (11.2,12.7)	1,606	870,093	18.7 (17.8,19.7)	8.1	76.4	(66.4, 86.4)
Insurance															
Medicaid	173	40,626	42.6 (36.2,48.9)	139	47,356	29.4 (24.5,34.2)	94	51,743	18.2 (14.5,21.8)	433	54,807	79 (71.6,86.4)	36.4	85.4	(66.2, 104.6)
Medicare	948	262,843	36.1 (33.8,38.4)	820	278,804	29.4 (27.4,31.4)	853	299,763	28.5 (26.5,30.4)	1,168	316,330	36.9 (34.8,39)	0.8	2.2	(-5.2, 9.6)
Private	449	847,442	5.3 (4.8,5.8)	482	837,009	5.8 (5.2,6.3)	585	876,007	6.7 (6.1,7.2)	870	906,023	9.6 (9,10.2)	4.3	81.1	(67.9, 94.3)
Uninsured	105	153,935	6.8 (5.5,8.1)	120	181,493	6.6 (5.4,7.8)	124	150,163	8.3 (6.8,9.7)	102	135,703	7.5 (6.1,9)	0.7	10.3	(-13.7, 34.3)
Total	1,707	1,328,234	12.5 (11.9,13.1)	1,616	1,383,448	11.5 (10.9,12)	1,711	1,412,937	11.9 (11.3,12.4)	2,679	1,445,012	18.2 (17.5,18.9)	5.7	45.6	(39, 52.2)

[a] Total # of ED Visits N=7,713

[b] All rates except Age-Specific and Insurance-Specific rates are age-standardized to the 2010 US Adult Population using data from the CDC Mortality Database

[c] The transition from ICD-9 CM/PCS to ICD-10 CM/PCS coding occured in third quarter of 2015. Estimates reported from years post-2015 were estimated using ICD-10 codes and may not be directly comparable to rates estimated pre-2015.

© 2022 Uppal TS et al. *JAMA Network Open.*

eTable 15. Rates of Diabetes-Specific Emergency Department Visits per 10,000 Adults in New Jersey; Years 2008-2017

	2008				2011				2014				2017				2017-2008		
**Variable**	**# of E.D. Visits**	**Denominator**	**Rate**	**(c.i.)**	**# of E.D. Visits**	**Denominator**	**Rate**	**(c.i.)**	**# of E.D. Visits**	**Denominator**	**Rate**	**(c.i.)**	**# of E.D. Visits**	**Denominator**	**Rate**	**(c.i.)**	**Absolute △**	**Percent △**	**(c.i.)**
Age																			
18-29	567	1,306,349	4.3	(4,4.7)	635	1,324,379	4.8	(4.4,5.2)	566	1,367,695	4.1	(3.8,4.5)	1,213	1,372,927	8.8	(8.3,9.3)	4.5	104.7	(90.9, 118.5)
30-44	1,496	1,862,521	8.0	(7.6,8.4)	1,620	1,783,584	9.1	(8.6,9.5)	1,529	1,753,846	8.7	(8.3,9.2)	2,951	1,749,689	16.9	(16.3,17.5)	8.9	111.2	(103.8, 118.6)
45-64	3,444	2,322,663	14.8	(14.3,15.3)	3,920	2,461,341	15.9	(15.4,16.4)	4,057	2,494,736	16.3	(15.8,16.8)	7,346	2,490,992	29.5	(28.8,30.2)	14.7	99.3	(94.6, 104)
65-74	1,263	539,915	23.4	(22.1,24.7)	1,316	589,674	22.3	(21.1,23.5)	1,423	676,027	21.0	(20,22.1)	2,202	739,520	29.8	(28.5,31)	6.4	27.4	(20.9, 33.9)
75+	1,902	604,153	31.5	(30.1,32.9)	1,740	621,296	28.0	(26.7,29.3)	1,730	634,566	27.3	(26,28.5)	2,467	675,978	36.5	(35.1,37.9)	5	15.9	(10.7, 21.1)
Sex																			
Female	4,107	3,426,778	11.3	(11,11.6)	4,300	3,527,273	11.5	(11.1,11.8)	4,197	3,591,661	10.9	(10.6,11.2)	7,370	3,641,398	19.1	(18.7,19.6)	7.8	69.0	(64.9, 73.1)
Male	4,565	3,208,823	14.6	(14.2,15)	4,931	3,253,001	15.2	(14.8,15.6)	5,108	3,335,209	15.2	(14.8,15.6)	8,809	3,387,708	25.6	(25.1,26.2)	11	75.3	(71.2, 79.4)
Race/Ethnicity																			
White, Non-Hispanic	2,775	838,203	36.5	(35.1,37.9)	2,938	840,695	36.4	(35.1,37.8)	2,821	869,873	33.5	(32.2,34.7)	5,538	886,765	63.3	(61.6,65)	26.8	73.4	(68.2, 78.6)
Black, Non-Hispanic	1,275	996,973	16.3	(15.3,17.2)	1,486	1,128,479	16.7	(15.8,17.6)	1,423	1,225,135	14.2	(13.4,15)	3,318	1,307,725	29.0	(28,30.1)	12.7	77.9	(70.7, 85.1)
Hispanic	4,067	4,229,385	8.6	(8.4,8.9)	4,312	4,131,094	9.3	(9,9.6)	4,372	4,087,664	9.4	(9.1,9.7)	6,011	4,025,066	13.5	(13.2,13.9)	4.9	57.0	(52.7, 61.3)
Insurance																			
Medicaid	642	290,635	22.1	(20.4,23.8)	933	361,594	25.8	(24.1,27.5)	1,498	462,056	32.4	(30.8,34.1)	3,521	584,094	60.3	(58.3,62.3)	38.2	172.9	(162.9, 182.9)
Medicare	3,774	1,250,959	30.2	(29.2,31.1)	3,820	1,311,515	29.1	(28.2,30.1)	4,003	1,415,311	28.3	(27.4,29.2)	5,745	1,522,964	37.7	(36.7,38.7)	7.5	24.8	(20.9, 28.7)
Private	2,414	4,139,238	5.8	(5.6,6.1)	2,400	4,009,016	6.0	(5.7,6.2)	2,617	4,102,112	6.4	(6.1,6.6)	4,692	4,249,595	11.0	(10.7,11.4)	5.2	89.7	(83.3, 96.1)
Uninsured	1,748	923,607	18.9	(18,19.8)	2,011	1,060,003	19.0	(18.1,19.8)	1,099	910,641	12.1	(11.4,12.8)	1,406	629,971	22.3	(21.2,23.5)	3.4	18.0	(11.2, 24.8)
Total	8,672	6,635,601	12.9	(12.6,13.1)	9,231	6,780,274	13.2	(13,13.5)	9,305	6,926,870	12.9	(12.7,13.2)	16,179	7,029,106	22.2	(21.9,22.6)	9.3	72.1	(69.2, 75)

[a] Total # of ED Visits N=43,387

[b] All rates except Age-Specific and Insurance-Specific rates are age-standardized to the 2010 US Adult Population using data from the CDC Mortality Database

[c] The transition from ICD-9 CM/PCS to ICD-10 CM/PCS coding occured in third quarter of 2015. Estimates reported from years post-2015 were estimated using ICD-10 codes and may not be directly comparable to rates estimated pre-2015.

© 2022 Uppal TS et al. *JAMA Network Open.*

eTable 16. Rates of Diabetes-Specific Emergency Department Visits per 10,000 Adults in New York; Years 2008-2016

	2008			2011			2014			2017			2017-2008		
**Variable**	**# of E.D. Visits**	**Denominator**	**Rate (c.i.)**	**# of E.D. Visits**	**Denominator**	**Rate (c.i.)**	**# of E.D. Visits**	**Denominator**	**Rate (c.i.)**	**# of E.D. Visits**	**Denominator**	**Rate (c.i.)**	**Absolute △**	**Percent △**	**(c.i.)**
Age															
18-29	1,498	3,330,436	4.5 (4.3,4.7)	1,713	3,382,395	5.1 (4.8,5.3)	1,714	3,449,034	5.0 (4.7,5.2)	3,172	3,406,325	9.3 (9.0,9.6)	4.8	106.9	(99.3, 114.5)
30-44	3,986	4,020,959	9.9 (9.6,10.2)	3,861	3,890,674	9.9 (9.6,10.2)	3,932	3,898,392	10.1 (9.8,10.4)	6,406	3,861,706	16.6 (16.2,17.0)	6.7	67.4	(63, 71.8)
45-64	8,597	5,141,374	16.7 (16.4,17.1)	9,634	5,253,266	18.3 (18,18.7)	10,559	5,283,866	20.0 (19.6,20.4)	16,070	5,273,615	30.5 (30,30.9)	13.8	82.2	(79.2, 85.2)
65-74	2,852	1,224,393	23.3 (22.4,24.2)	3,128	1,290,368	24.2 (23.4,25.1)	3,479	1,486,157	23.4 (22.6,24.2)	4,970	1,586,994	31.3 (30.5,32.2)	8.0	34.5	(30.1, 38.9)
75+	3,626	1,376,299	26.4 (25.5,27.2)	3,735	1,372,131	27.2 (26.3,28.1)	3,913	1,410,167	27.8 (26.9,28.6)	4,976	1,443,619	34.5 (33.5,35.4)	8.1	30.8	(26.7, 34.9)
Sex															
Female	9,856	7,874,358	12.1 (11.9,12.4)	10,308	7,950,682	12.5 (12.3,12.8)	10,841	8,106,141	12.9 (12.6,13.1)	16,496	8,119,687	19.7 (19.4,20.0)	7.5	61.9	(59.3, 64.5)
Male	10,703	7,219,103	15.3 (15,15.6)	11,763	7,238,152	16.6 (16.3,16.9)	12,756	7,421,475	17.4 (17.1,17.7)	19,098	7,452,572	25.8 (25.4,26.2)	10.5	68.7	(66.1, 71.3)
Race/Ethnicity															
Hispanic	9,406	9,355,249	9.4 (9.2,9.6)	9,263	9,098,525	9.5 (9.3,9.7)	9,913	9,068,059	10.1 (9.9,10.3)	12,538	8,958,153	13.2 (13.0,13.4)	3.8	40.4	(37.7, 43.1)
Hispanic	6,240	2,129,649	31.3 (30.5,32.1)	6,885	2,136,385	33.6 (32.8,34.4)	7,019	2,194,101	33.0 (32.2,33.8)	11,607	2,203,763	53.7 (52.7,54.6)	22.4	71.4	(68, 74.8)
Hispanic	2,689	2,308,197	14.1 (13.5,14.6)	3,092	2,514,280	14.9 (14.4,15.5)	3,848	2,661,023	16.9 (16.4,17.5)	6,534	2,723,870	26.9 (26.2,27.5)	12.8	90.6	(85.4, 95.8)
Rural/Urban															
Rural	2,117	1,243,723	16.6 (15.9,17.4)	2,377	1,250,394	18.1 (17.4,18.9)	2,337	1,235,524	17.9 (17.2,18.7)	2,839	1,214,296	22.5 (21.6,23.3)	5.8	35.0	(29.4, 40.6)
Urban	18,442	13,849,738	13.3 (13.2,13.5)	19,694	13,938,440	14.1 (13.9,14.3)	21,260	14,292,092	14.8 (14.6,15.0)	32,755	14,357,963	22.6 (22.4,22.9)	9.3	69.6	(67.6, 71.6)
Insurance															
Medicaid	2,794	1,351,364	20.7 (19.9,21.4)	6,021	1,760,708	34.2 (33.3,35.1)	6,903	2,043,074	33.8 (33.0,34.6)	12,409	2,318,372	53.5 (52.6,54.5)	32.8	158.8	(153.9, 163.7)
Medicare	6,345	2,887,945	22.0 (21.4,22.5)	8,468	2,963,084	28.6 (28,29.2)	9,612	3,221,167	29.8 (29.2,30.4)	12,523	3,348,570	37.4 (36.7,38.1)	15.4	70.2	(66.9, 73.5)
Private	8,236	8,727,751	9.4 (9.2,9.6)	4,432	8,288,813	5.3 (5.2,5.5)	4,258	8,581,836	5.0 (4.8,5.1)	6,449	8,688,671	7.4 (7.2,7.6)	-2.0	-21.4	(-23.8, -19)
Uninsured	2,913	2,019,786	14.4 (13.9,15.0)	2,800	2,061,120	13.6 (13.1,14.1)	2,334	1,569,929	14.9 (14.3,15.5)	3,617	1,104,604	32.8 (31.7,33.8)	18.3	127.1	(120.2, 134)
Total	20,559	15,093,461	13.6 (13.4,13.8)	22,071	15,188,834	14.5 (14.3,14.7)	23,597	15,527,616	15.0 (14.8,15.2)	35,594	15,572,259	22.6 (22.4,22.8)	9.0	65.8	(63.9, 67.7)

[a] Total # of ED Visits N=101,821

[b] All rates except Age-Specific and Insurance-Specific rates are age-standardized to the 2010 US Adult Population using data from the CDC Mortality Database

[c] The transition from ICD-9 CM/PCS to ICD-10 CM/PCS coding occured in third quarter of 2015. Estimates reported from years post-2015 were estimated using ICD-10 codes and may not be directly comparable to rates estimated pre-2015.

© 2022 Uppal TS et al. *JAMA Network Open.*

eTable 17. Rates of Diabetes-Specific Emergency Department Visits per 10,000 Adults in Utah; Years 2008-2016

	2008			2011			2014			2017			2017-2008		
**Variable**	**# of E.D. Visits**	**Denominator**	**Rate (c.i.)**	**# of E.D. Visits**	**Denominator**	**Rate (c.i.)**	**# of E.D. Visits**	**Denominator**	**Rate (c.i.)**	**# of E.D. Visits**	**Denominator**	**Rate (c.i.)**	**Absolute △**	**Percent △**	**(c.i.)**
Age															
18-29	252	590,462	4.3 (3.7,4.8)	173	545,856	3.2 (2.7,3.6)	193	547,583	3.5 (3.0,4.0)	568	586,594	9.7 (8.9,10.5)	5.4	126.7	(107.7, 145.7)
30-44	379	516,453	7.3 (6.6,8.1)	314	567,614	5.5 (4.9,6.1)	440	610,255	7.2 (6.6,7.9)	886	640,622	13.8 (12.9,14.7)	6.5	88.4	(75, 101.8)
45-64	720	538,738	13.4 (12.4,14.3)	731	563,825	13.0 (12.0,13.9)	808	585,497	13.8 (12.9,14.8)	1,376	614,875	22.4 (21.2,23.6)	9	67.5	(57.9, 77.1)
65-74	263	121,334	21.7 (19.1,24.3)	259	130,992	19.8 (17.4,22.2)	295	159,439	18.5 (16.4,20.6)	450	184,712	24.4 (22.1,26.6)	2.7	12.4	(-1, 25.8)
75+	365	124,037	29.4 (26.4,32.5)	307	127,571	24.1 (21.4,26.8)	283	134,979	21.0 (18.5,23.4)	418	150,203	27.8 (25.2,30.5)	-1.6	-5.4	(-16.9, 6.1)
Sex															
Female	932	952,000	10.6 (9.9,11.3)	888	971,560	10.0 (9.3,10.6)	916	1,024,043	9.6 (9.0,10.2)	1,761	1,092,186	16.8 (16.0,17.6)	6.1	57.7	(49.4, 66)
Male	1,047	939,024	13.2 (12.4,14.0)	896	964,298	10.8 (10.1,11.5)	1,103	1,013,710	12.1 (11.4,12.9)	1,937	1,084,820	19.3 (18.4,20.1)	6.1	46.3	(38.7, 53.9)
Race/Ethnicity															
Hispanic	1,625	1,588,818	11.2 (10.7,11.8)	1,404	1,592,695	9.5 (9.0,10.0)	1,461	1,657,244	9.3 (8.8,9.8)	2,844	1,745,144	16.8 (16.2,17.5)	5.6	50.0	(43.7, 56.3)
Hispanic	34	16,936	24.9 (15.5,34.2)	35	15,819	30.0 (17.0,43.1)	34	20,701	19.4 (12.0,26.9)	136	24,066	65.2 (52.0,78.4)	40.3	162.1	(107.3, 216.9)
Hispanic	228	193,733	17.7 (14.9,20.4)	242	224,168	18.3 (15.6,21.1)	344	242,961	22.0 (19.3,24.7)	380	273,160	18.2 (16.1,20.2)	0.5	2.8	(-13.6, 19.2)
Rural/Urban															
Rural	315	313,916	10.3 (9.2,11.5)	294	322,547	9.0 (8.0,10.1)	302	272,165	11.3 (10.0,12.6)	482	289,750	17.0 (15.5,18.5)	6.6	64.3	(48.7, 79.9)
Urban	1,664	1,577,108	12.2 (11.62,12.82)	1,490	1,613,311	10.8 (10.2,11.4)	1,717	1,765,588	10.8 (10.2,11.3)	3,216	1,887,256	18.0 (17.4,18.7)	5.8	47.7	(41.7, 53.7)
Insurance															
Medicaid	282	61,304	46.0 (40.6,51.3)	315	79,256	39.8 (35.3,44.3)	343	68,354	50.2 (44.8,55.5)	559	78,870	70.8 (64.9,76.8)	24.9	54.1	(39.4, 68.8)
Medicare	708	272,464	26.0 (24.1,27.9)	709	286,559	24.7 (22.9,26.6)	848	329,176	25.8 (24.0,27.5)	1,305	362,242	36.0 (34.1,38.0)	10	38.6	(29.7, 47.5)
Private	772	1,233,040	6.3 (5.81,6.7)	530	1,213,744	4.4 (4.0,4.7)	590	1,323,294	4.5 (4.1,4.8)	1,165	1,480,402	7.9 (7.4,8.3)	1.6	25.7	(17.1, 34.3)
Uninsured	187	296,240	6.3 (5.41,7.24)	180	331,214	5.4 (4.7,6.2)	201	285,740	7.0 (6.0,8.0)	488	223,794	21.8 (19.9,23.8)	15.5	244.7	(216.1, 273.3)
Total	1,979	1,891,024	11.8 (11.3,12.4)	1,784	1,935,858	10.4 (9.9,10.9)	2,019	2,037,753	10.8 (10.3,11.3)	3,698	2,177,006	18.0 (17.4,18.6)	6.1	51.9	(46.3, 57.5)

[a] Total # of ED Visits N=9,480

[b] All rates except Age-Specific and Insurance-Specific rates are age-standardized to the 2010 US Adult Population using data from the CDC Mortality Database

[c] The transition from ICD-9 CM/PCS to ICD-10 CM/PCS coding occured in third quarter of 2015. Estimates reported from years post-2015 were estimated using ICD-10 codes and may not be directly comparable to rates estimated pre-2015.

© 2022 Uppal TS et al. *JAMA Network Open.*

eTable 18. Rates of Diabetes-Specific Emergency Department Visits per 10,000 Adults in Vermont; Years 2008-2017

	2008				2011				2014				2017				2017-2008		
**Variable**	**# of E.D. Visits**	**Denominator**	**Rate**	**(c.i.)**	**# of E.D. Visits**	**Denominator**	**Rate**	**(c.i.)**	**# of E.D. Visits**	**Denominator**	**Rate**	**(c.i.)**	**# of E.D. Visits**	**Denominator**	**Rate**	**(c.i.)**	**Absolute △**	**Percent △**	**(c.i.)**
Age																			
18-29	77	99,268	7.8	(6,9.5)	76	99,277	7.7	(5.9,9.4)	73	101,792	7.2	(5.5,8.8)	102	102,030	10.0	(8.1,11.9)	2.2	28.2	(-0.3, 56.7)
30-44	121	115,418	10.5	(8.6,12.4)	104	112,475	9.2	(7.5,11)	114	110,046	10.4	(8.5,12.3)	175	106,355	16.5	(14,18.9)	6.0	57.1	(32.6, 81.6)
45-64	311	191,266	16.3	(14.5,18.1)	285	193,415	14.7	(13,16.4)	292	186,455	15.7	(13.9,17.5)	421	182,085	23.1	(20.9,25.3)	6.8	41.7	(27.3, 56.1)
65-74	118	44,410	26.6	(21.8,31.4)	107	47,543	22.5	(18.2,26.8)	145	58,175	24.9	(20.9,29)	168	63,713	26.4	(22.4,30.4)	-0.2	-0.8	(-20.2, 18.6)
75+	179	42,854	41.8	(35.7,47.9)	143	46,933	30.5	(25.5,35.5)	128	48,020	26.7	(22,31.3)	187	50,412	37.1	(31.8,42.4)	-4.7	-11.2	(-27.4, 5)
Sex																			
Female	365	254,338	13.7	(12.3,15.1)	316	254,641	11.7	(10.4,13)	344	260,641	12.5	(11.1,13.8)	518	259,493	19.0	(17.4,20.7)	5.3	38.7	(25, 52.4)
Male	441	238,878	18.3	(16.6,20)	399	245,002	15.9	(14.4,17.5)	408	243,847	16.0	(14.5,17.6)	535	245,102	20.9	(19.2,22.7)	2.6	14.2	(2.7, 25.7)
Rural/Urban																			
Rural	460	331,730	13.1	(11.9,14.3)	430	329,683	12.1	(11,13.3)	465	330,868	13.1	(11.9,14.4)	690	330,050	19.7	(18.2,21.2)	6.6	50.4	(37.8, 63)
Urban	346	161,486	22.1	(19.8,24.4)	285	169,960	17.4	(15.3,19.4)	287	173,620	17.0	(15,18.9)	363	174,545	20.8	(18.6,22.9)	-1.3	-5.9	(-18.1, 6.3)
Insurance																			
Medicaid	139	45,815	30.3	(25.3,35.4)	159	56,954	27.9	(23.6,32.3)	175	65,776	26.6	(22.7,30.5)	269	69,271	38.8	(34.2,43.5)	8.5	28.1	(8.8, 47.4)
Medicare	406	102,640	39.6	(35.7,43.4)	351	106,997	32.8	(29.4,36.2)	379	124,297	30.5	(27.4,33.6)	534	128,549	41.5	(38,45.1)	1.9	4.8	(-6.4, 16)
Private	203	291,962	7.0	(6,7.9)	171	286,474	6.0	(5.1,6.9)	154	281,172	5.5	(4.6,6.3)	197	280,782	7.0	(6,8)	0.0	0.0	(-16.7, 16.7)
Uninsured	54	47,088	11.5	(8.4,14.5)	23	42,758	5.4	(3.2,7.6)	23	28,058	8.2	(4.8,11.5)	41	20,787	19.7	(13.7,25.8)	8.2	71.3	(21.2, 121.4)
Total	806	493,216	15.9	(14.8,17)	715	499,643	13.7	(12.7,14.7)	752	504,488	14.1	(13.1,15.2)	1,053	504,595	19.9	(18.7,21.1)	4.0	25.2	(16.4, 34)

[a] Total # of ED Visits N=3,326

[b] All rates except Age-Specific and Insurance-Specific rates are age-standardized to the 2010 US Adult Population using data from the CDC Mortality Database

[c] The transition from ICD-9 CM/PCS to ICD-10 CM/PCS coding occured in third quarter of 2015. Estimates reported from years post-2015 were estimated using ICD-10 codes and may not be directly comparable to rates estimated pre-

© 2022 Uppal TS et al. *JAMA Network Open.*

eTable 19. Average Rates of Diabetes-Specific Emergency Department Visits From Pooled State Data by Race, Rural/Urban, and Insurance; Years 2008-2017

	2008	2011	2014	2016 / 2017*
	Rate (95% CI)	Rate (95% CI)	Rate (95% CI)	Rate (95% CI)
Race				
Black, non-Hispanic	32.4 (31.9,33)	37.1 (36.7,37.6)	37.3 (36.9,37.7)	63.3 (62.7,63.8)
Hispanic	14.2 (13.8,14.7)	13.3 (13,13.5)	15.7 (15.5,16)	25.9 (25.6,26.2)
White, non-Hispanic	10.7 (10.6,10.9)	12.1 (12,12.2)	12.9 (12.8,13)	19.7 (19.6,19.8)
Rural/Urban				
Rural	20.2 (19.9,20.5)	21.1 (20.8,21.4)	21 (20.7,21.3)	31.1 (30.7,31.4)
Urban	14.1 (14,14.2)	14.9 (14.8,15)	16.4 (16.3,16.5)	26.4 (26.3,26.5)
Insurance				
Medicaid	36.4 (35.7,37)	41.8 (41.2,42.4)	44.2 (43.7,44.8)	74.1 (73.4,74.8)
Medicare	30.5 (30.2,30.8)	31.9 (31.6,32.2)	31.3 (31,31.6)	41.3 (41,41.6)
Private	7.2 (7.1,7.3)	6 (6,6.1)	6.7 (6.6,6.7)	10.9 (10.8,11)
Uninsured	14 (13.8,14.2)	14.1 (13.9,14.4)	14.7 (14.5,15)	32.2 (31.7,32.6)

Rates were generated using average ED visits aggregated from pooled SEDD data from states AZ, FL, IA, KY, MD, NC, NE, NJ, NY, UT, VT. Population estimates generated from pooled state populations estimated from the IPUMS USA American Community Survey for corresponding years.

All rates except insurance were age-standardized to the 2010 US Adult Population using population estimates available from the CDC Mortality Database

*Average rates calculated from an average ED utilization rates from 2016 and 2017 estimates, as 2017 data was not available for select states.

**Average use across states for Race estimates do not include NE and VT due to missing race variable and low n of numerator and denominator estimates

**Average use across states for Rural/Urban estimates do not include NJ due to lack of rural counties as defined by USDA supplied rural/urban variable

eFigure 3. Age-Adjusted Rates of All-Cause Diabetes Emergency Department Use Among US Adults by Race/Ethnicity; Years 2008-2017

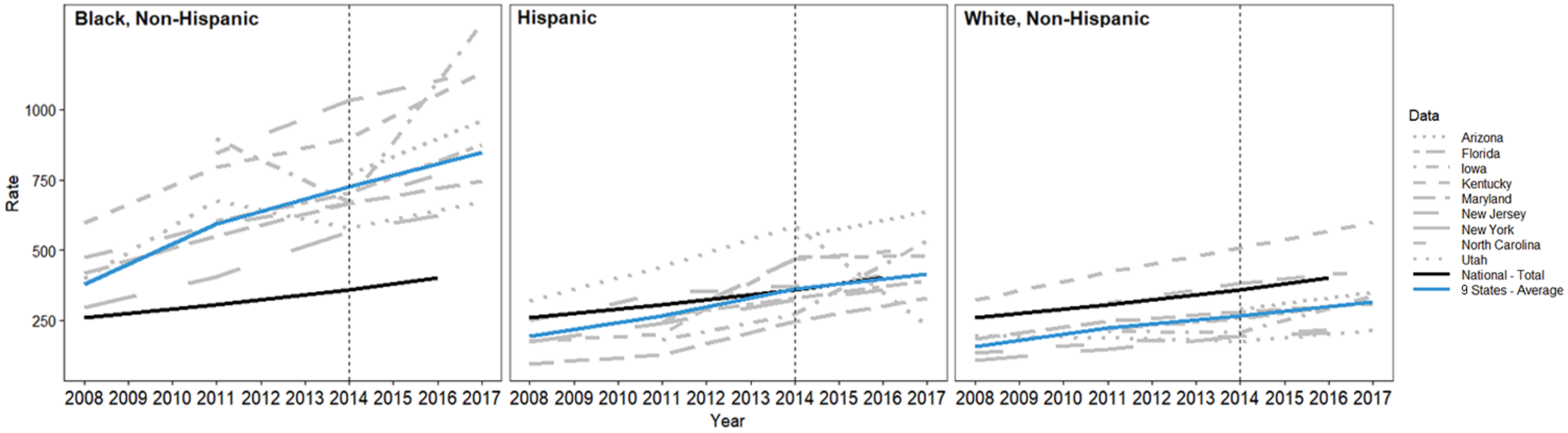

ED utilization rates were calculated using numerator data from the HCUP National Emergency Department Sample and State Emergency Department Databases and denominator data from the IPUMS USA American Community Survey from years 2008-2017. All rates are age-standardized to the national 2010 US adult population using data from the CDC Mortality Database.

Two benchmark lines are highlighted, the black National-Total line shoes national ED use across all US adults, regardless of race/ethnicity and the blue 9 States-Average line shows average ED use across all states by race/ethnicity group.

Rates were not reported for state data with inconsistently coded race/ethnicity variables and/or sub-group estimates with estimates n≤10 events or RSE >= 30%.
Nevada did not supply race/ethnicity data.

The dotted vertical line delineates the point within our datasets that the data transitioned from rates generated using ICD-9-CM codes to ICD-10-CM codes. Rates post-transition may not be directly comparable to rates prior to the transition.

© 2022 Uppal TS et al. *JAMA Network Open.*

eFigure 4. Age-Adjusted Rates of All-Cause Diabetes Emergency Department Use Among US Adults by Rural Status; Years 2008-2017

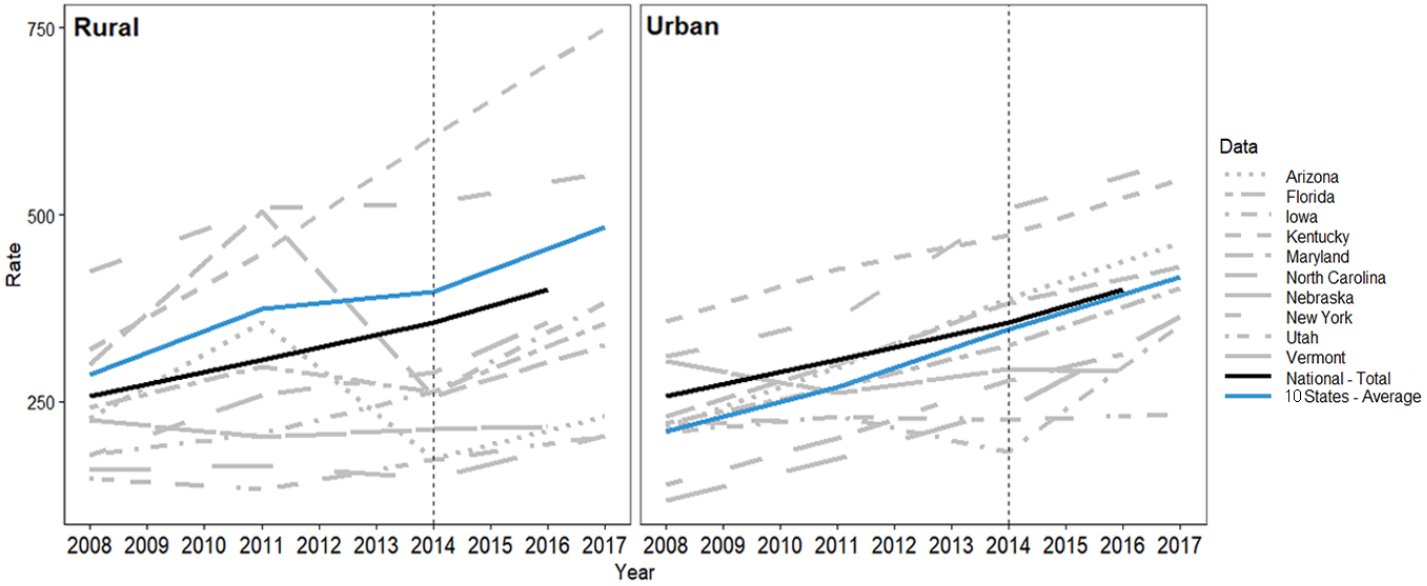

ED Utilization rates were calculated using numerator data from the HCUP National Emergency Department Sample and State Emergency Department Databases and denominator data from the IPUMS USA American Community Survey from years 2008-2017. All rates are age-standardized to the national 2010 US adult population using data from the CDC Mortality Database.

Two benchmark lines are highlighted: the black National-Total line shows national ED use across all US adults, regardless of rural/urban status and the blue 10 States-Average line shows average ED use across all states by rural/urban status.

Rates were not reported for state data with inconsistently coded race/ethnicity variables and/or sub-group estimates with n≤10 events or RSE>30%. The denominator rural/urban variable did not allocate any rural counties in New Jersey.

The dotted vertical line delineates the point within our data that the data transitioned from rates generated using ICD-9 codes to ICD-10 codes. Rates post-transition may not be directly comparable to rates prior to the transition.

© 2022 Uppal TS et al. *JAMA Network Open.*

eFigure 5. Insurance-Specific Rates of Diabetes-Specific Emergency Department Use Among US Adults; Years 2008-2017

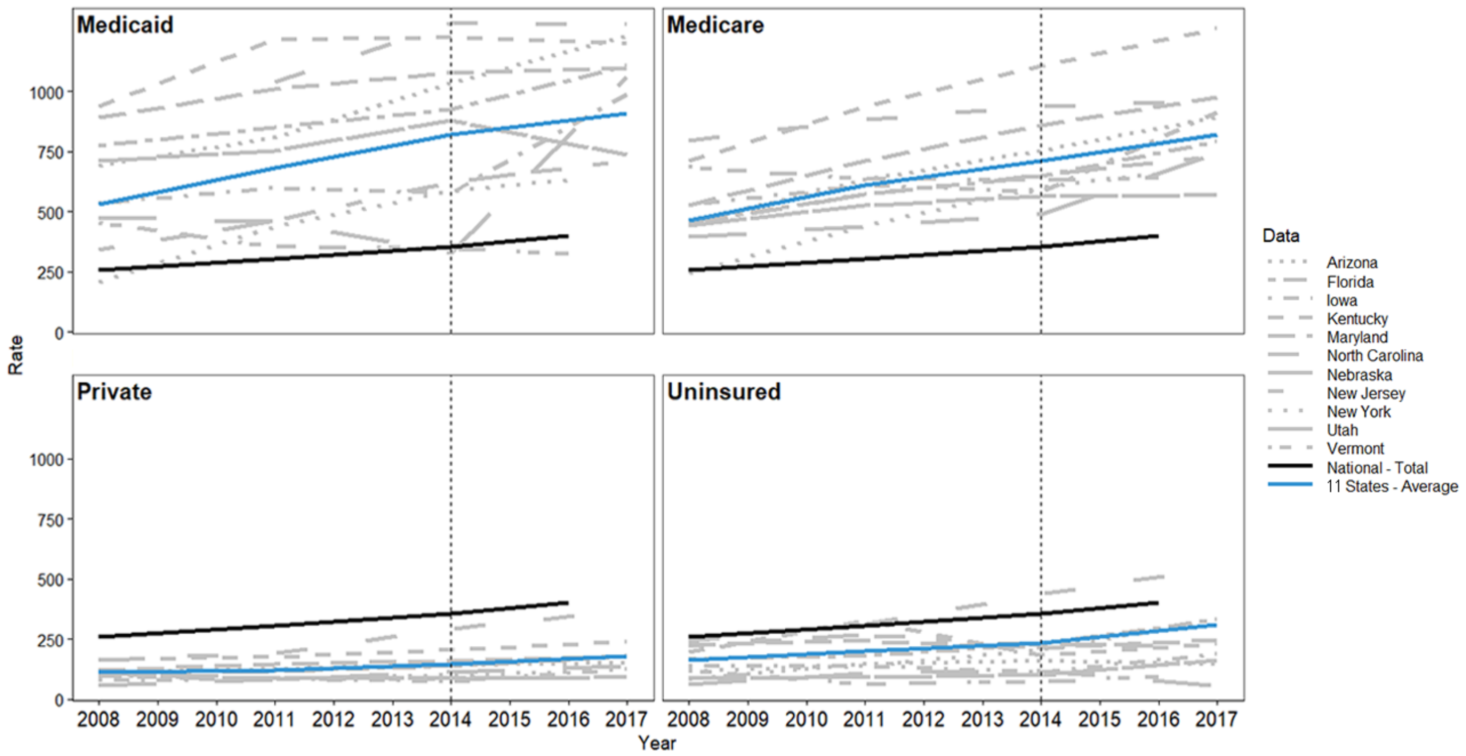

ED utilization rates were calculated using numerator data from the HCUP National Emergency Department Sample and State Emergency Department Databases and denominator data from from the IPUMS USA American Community Survey from years 2008-2017.

Two benchmark lines are highlighted: the National-Total line shows national ED use across all US adults, regardless of insurance status and the 11 States-Average line shows average ED use across all states by insurance group.

The dotted vertical line delineates the point within our datasets that the data transitioned from rates generated using ICD-9-CM codes to ICD-10-CM codes. Rates post-transition may not be directly comparable to rates prior to the transition.

© 2022 Uppal TS et al. *JAMA Network Open.*

eTable 20. Proportion of Diabetes-Specific Emergency Department Discharges by Number of Diagnoses on Record; Years 2008-2016[a]

	2008	2011	2014	2016
# of Diagnoses	n (95% CI)			
1-3	0.261	0.203	0.171	0.233
	(0.259, 0.264)	(0.201, 0.205)	(0.169, 0.173)	(0.231, 0.234)
4-7	0.322	0.304	0.284	0.318
	(0.320, 0.324)	(0.302, 0.306)	(0.282, 0.287)	(0.316, 0.320)
8-11	0.215	0.222	0.216	0.186
	(0.213, 0.217)	(0.220, 0.224)	(0.214, 0.218)	(0.184, 0.188)
11-14	0.087	0.102	0.111	0.090
	(0.086, 0.088)	(0.100, 0.103)	(0.109, 0.112)	(0.088, 0.091)
≥15	0.115	0.169	0.218	0.174
	(0.113, 0.116)	(0.167, 0.171)	(0.216, 0.220)	(0.172, 0.176)

[a] Proportions calculated from National Emergency Department Sample data, years 2008, 2011, 2014, 2016. Diabetes-specific visits include all visits with a principal diagnosis of a diabetes-specific condition/complication.

To evaluate evidence of upcoding net of changes in comorbidity among patients with diabetes, we examined the number of diagnoses per diabetes-specific discharges using the NEDS. Diabetes-specific discharges are for a defined set of primary conditions caused by diabetes complications that can be avoided with quality primary care. Since the criteria for these episodes of care remains constant over time and are the principal diagnosis (primary medical reason) for the patient seeking care, changes in the volume of additional diagnostic codes for these patients provide a measure of upcoding with less confounding changes in population health. We found that the proportion of discharges with 1-3 diagnoses on record decreased from 26.1% (95 CI: 0.259-0.264) in 2008 to 17.1% (95 CI: 0.169-0.173) in 2014. While there were relatively smaller fluctuations among the intermediary groups of 4-7, 8-11, and 11-14 diagnoses over the period, there was a pronounced increase in the proportion with ≥15 diagnoses over the period studied, which increased from 11.5% (95 CI: 0.113-0.116) to 21.8% (95 CI: 0.216-0.220) in 2014.

These results indicate the presence of upcoding, with the strongest evidence observed among decreases in the 1-3 group and increases in the ≥15 diagnoses group. Upcoding is likely to have affected our all-cause diabetes rates, which includes all discharges with any diabetes diagnosis on records.

However, our diabetes-specific rates only examined discharges with a principal diagnosis of a diabetes-specific complication, so while these discharges have additional diagnoses, the underlying numerator would not be impacted. Since we cannot directly attribute the reasons for difference in trends for all-cause ED use and diabetes-specific ED use, we focus our discussion on the variation observed between states and sociodemographic groups for diabetes-specific complications. Further, our examination of state- and sociodemographic variation in diabetes-specific use would also be robust to upcoding.

© 2022 Uppal TS et al. *JAMA Network Open.*

eReferences.

1. Ma Y, Zhang W, Lyman S, Huang Y. The HCUP SID imputation project: improving statistical inferences for health disparities research by imputing missing race data. *Health services research.* 2018;53(3):1870-1889.
2. Steven Ruggles SF, Ronald Goeken, Josiah Grover, Erin Meyer, Jose Pacas and Matthew Sobek. PUMS USA: Version 10.0 [dataset]. IPUMS. Published 2020. Accessed2020.
3. Molenberghs G, Verbeke G. *Linear mixed models for longitudinal data.* Springer; 2000.
4. Gelman A, Hill J. *Data analysis using regression and multilevel/hierarchical models.* Cambridge university press; 2006.
5. Little RJ, Rubin DB. *Statistical analysis with missing data.* Vol 793: John Wiley & Sons; 2019.
6. Buuren Sv, Groothuis-Oudshoorn K. Mice: Multivariate imputation by chained equations in RJ Stat. Softw. 45. In:2011.
7. Van Buuren S. *Flexible imputation of missing data.* CRC press; 2018.
8. Agency For Healthcare Research and Quality (AHRQ). Prevention Quality Indicators. AHRQ - Quality Indicators Web site. https://www.qualityindicators.ahrq.gov/Modules/PQI_TechSpec_ICD10_v2020.aspx. Published 2020. Accessed 2020, January.
9. Tseng C-L, Soroka O, Pogach LM. An expanded prevention quality diabetes composite: Quantifying the burden of preventable hospitalizations for older adults with diabetes. *Journal of Diabetes and its Complications.* 2018;32(5):458-464.
10. Centers for Medicare & Medicaid Services (CMS). Medicare & Other Health Benefits: Your Guide to Who Pays First. U.S. DEPARTMENT OF HEALTH AND HUMAN SERVICES. https://www.medicare.gov/Pubs/pdf/02179-medicare-coordination-benefits-payer.pdf. Published 2020. Accessed 2020, August.
11. Barrett M, Lopez-Gonzalez L, Hines A, Andrews R, Jiang J. *An Examination of Expected Payer Coding in HCUP Databases.* U.S. Agency for Healthcare Research and Quality; 2014/12/17/ 2014. 2014-03.
12. Economic Research Service (ERS). Rural Classifications. United States Department of Agriculture. https://www.ers.usda.gov/topics/rural-economy-population/rural-classifications/. Published 2019. Updated October 23, 2019. Accessed.
13. Barrett M, Coffey, R, Houchens, R, Heslin, K, Moles, E, Coenen, N. *Methods Applying AHRQ Quality Indicators to Healthcare Cost and Utilization Project (HCUP) Data for the 2017 National Healthcare Quality and Disparities Report (QDR).* U.S. Agency for Healthcare Research and Quality; May 11, 2018 2018.
14. Lumley T. Analysis of complex survey samples. *J Stat Softw.* 2004;9(1):1-19.
15. LaMantia MA, Lane KA, Tu W, Carnahan JL, Messina F, Unroe KT. Patterns of emergency department use among long-stay nursing home residents with differing levels of dementia severity. *Journal of the American Medical Directors Association.* 2016;17(6):541-546.
16. Patel PB, Vinson DR, Gardner MN, et al. Impact of emergency physician–provided patient education about alternative care venues. *Am J Manag Care.* 2018;24(5):225.
17. Centers for Disease Control and Prevention National Center for Health Statistics. Underlying Cause of Death 1999-2018 on CDC WONDER Online Database, released in 2019. Data are from the Multiple Cause of Death Files, 1999-2018, as compiled from data provided by the 57 vital statistics jurisdictions through the Vital Statistics Cooperative Program. In: Data are from the Multiple Cause of Death Files, 1999-2017, as compiled from data provided by the 57 vital statistics jurisdictions through the Vital Statistics Cooperative Program; 2018.

© 2022 Uppal TS et al. *JAMA Network Open.*

18. Roalfe AK, Holder RL, Wilson S. Standardisation of rates using logistic regression: a comparison with the direct method. *BMC Health Services Research.* 2008;8(1):275.
19. Gibson T, Casto A, Young J, Karnell L, Coenen N. *Impact of ICD-10-CM/PCS on Research Using Administrative Databases Report # 2016-02.* U.S. Agency for Healthcare Research and Quality; July 25, 2016 2016.
20. Newman SC. *Biostatistical methods in epidemiology.* Wiley Online Library; 2001.

© 2022 Uppal TS et al. *JAMA Network Open.*
